# Impact of pregnancy related hormones on drug metabolizing enzyme and transport protein concentrations in human hepatocytes

**DOI:** 10.3389/fphar.2022.1004010

**Published:** 2022-09-21

**Authors:** Muluneh M. Fashe, John K. Fallon, Taryn A. Miner, Jacqueline B. Tiley, Philip C. Smith, Craig R. Lee

**Affiliations:** ^1^ Division of Pharmacotherapy and Experimental Therapeutics, UNC Eshelman School of Pharmacy, University of North Carolina at Chapel Hill, Chapel Hill, NC, United States; ^2^ Division of Pharmacoengineering and Molecular Pharmaceutics, UNC Eshelman School of Pharmacy, University of North Carolina at Chapel Hill, Chapel Hill, NC, United States

**Keywords:** pregnanacy, targeted proteomics, uridine diphosphate glucuronosyltransferases (UGTs), carboxylesterase, flavin monooxygenase, drug transporters, pregnancy hormones, hepatic drug disposition

## Abstract

Pregnancy alters the disposition and exposure to multiple drugs indicated for pregnancy-related complications. Previous *in vitro* studies have shown that pregnancy-related hormones (PRHs) alter the expression and function of certain cytochrome P450s (CYPs) in human hepatocytes. However, the impact of PRHs on hepatic concentrations of non-CYP drug-metabolizing enzymes (DMEs) and transport proteins remain largely unknown. In this study, sandwich-cultured human hepatocytes (SCHH) from five female donors were exposed to vehicle or PRHs (estrone, estradiol, estriol, progesterone, cortisol, and placental growth hormone), administered individually or in combination, across a range of physiologically relevant PRH concentrations for 72 h. Absolute concentrations of 33 hepatic non-CYP DMEs and transport proteins were quantified in SCHH membrane fractions using a quantitative targeted absolute proteomics (QTAP) isotope dilution nanoLC-MS/MS method. The data revealed that PRHs altered the absolute protein concentration of various DMEs and transporters in a concentration-, isoform-, and hepatocyte donor-dependent manner. Overall, eight of 33 (24%) proteins exhibited a significant PRH-evoked net change in absolute protein concentration relative to vehicle control (ANOVA *p* < 0.05) across hepatocyte donors: 1/11 UGTs (9%; UGT1A4), 4/6 other DMEs (67%; CES1, CES2, FMO5, POR), and 3/16 transport proteins (19%; OAT2, OCT3, P-GP). An additional 8 (24%) proteins (UGT1A1, UGT2B4, UGT2B10, FMO3, OCT1, MRP2, MRP3, ENT1) exhibited significant PRH alterations in absolute protein concentration within at least two individual hepatocyte donors. In contrast, 17 (52%) proteins exhibited no discernable impact by PRHs either within or across hepatocyte donors. Collectively, these results provide the first comprehensive quantitative proteomic evaluation of PRH effects on non-CYP DMEs and transport proteins in SCHH and offer mechanistic insight into the altered disposition of drug substrates cleared by these pathways during pregnancy.

## Introduction

Medication use during pregnancy is on the rise. Approximately 80% of pregnant individuals use at least one medication during pregnancy, and about 30% are prescribed multiple medications (Ayad and Costantine, 2015; Haas et al., 2018). Although accumulating evidence demonstrates that the pharmacokinetics and effects of many drugs are altered during pregnancy, most drugs prescribed to pregnant individuals lack pregnancy-specific efficacy, safety, and dosing information based on studies conducted in this vulnerable population (Gonzalez et al., 2015; Koren and Pariente, 2018). Pharmacokinetic modeling studies have suggested that pregnancy-associated alterations in hepatic clearance are a key driver of alterations in systemic drug exposure and clearance for certain medications (Dallmann et al., 2018a; Dallmann et al., 2018b; Mulrenin et al., 2021). However, the key factors and pathways underlying altered hepatic drug disposition during pregnancy remain poorly understood and require further study to develop more precise medication selection and dosing recommendations for pregnant patients.

Notably, the synthesis and secretion of pregnancy-related hormones (PRHs) including estrogens, progesterone (P4), cortisol (CRT), and growth hormones increase by several fold during gestation (Newbern and Freemark, 2011). PRHs can activate their natural receptors including estrogen receptors (ERs), progesterone receptors (PRs), and glucocorticoid receptor (GR) or key xenosensors such as pregnane X receptor (PXR) and constitutive androstane receptor (CAR), which are known regulators of drug metabolizing enzyme (DME) and transport protein expression (Kawamoto et al., 2000; Xue et al., 2007; Brouwer et al., 2022). Thus, it has been hypothesized that increased secretion of PRHs during pregnancy drives changes in the pharmacokinetics of several drugs through altered expression of hepatic DMEs (Jeong, 2010; Isoherranen and Thummel, 2013). Studies have used cultured primary human hepatocytes exposed to PRHs as an experimental model system to investigate the impact of PRHs on DME expression and function (Jeong and Stika, 2020). Using this experimental model, several studies have demonstrated that PRHs alter the expression and function of certain cytochrome P450s (CYPs) in human hepatocytes (Choi et al., 2013; Dickmann and Isoherranen, 2013; Papageorgiou et al., 2013; Zhang et al., 2015; Khatri et al., 2021b). Most notably, PRHs significantly increase CYP3A4 and CYP2B6 expression and metabolic activity, which is consistent with and provides mechanistic insight into observed increases of CYP3A4- and CYP2B6-mediated metabolism and clearance of drugs in human pregnancy (Dickmann and Isoherranen, 2013; Isoherranen and Thummel, 2013; Tasnif et al., 2016; Mulrenin et al., 2021).

In contrast to a series of prior studies focused on CYPs, the impact of PRH on the expression and function of key non-CYP phase I DMEs (most notably carboxyltransferases (CESs) and flavin monooxygenases (FMOs)), phase II DMEs (most notably uridine 5′-diphospho-glucuronosyltransferase (UGTs)), and hepatic solute carrier (SLC) and ATP binding cassette (ABC) transport proteins have largely been unstudied. Numerous drug substrates of these hepatic pathways are frequently prescribed to pregnant individuals, including labetalol for hypertension (UGT1A1, UGT2B7), lamotrigine for seizure disorders (UGT1A4), oseltamivir for influenza (CES1), and ampicillin for infection (MRP2) (Shi et al., 2006; Jeong et al., 2008; Chen et al., 2009; Gupta et al., 2020). Although prior studies have evaluated PRH effects on non-CYP DME expression, these studies have been limited by use of stable cell lines or rodent hepatocytes, exposure to select individual PRHs, and evaluation of a select few isoforms at the mRNA level (Coecke et al., 1998; Chen et al., 2012; Fortin et al., 2013; Liao et al., 2018; Wu et al., 2018). Moreover, PRH effects on hepatic transport protein expression is largely unknown. Thus, there remains a substantial gap in knowledge regarding the presence and magnitude of PRH effects on protein concentrations across a comprehensive panel of key non-CYP phase I and II DMEs and hepatic transport proteins in primary human hepatocytes under the same experimental conditions.

Therefore, the primary objectives of the current study were to *1*) quantify baseline absolute concentrations of multiple non-CYP phase I and II DMEs and transport proteins in sandwich-cultured human female hepatocytes (SCHH) using quantitative targeted absolute proteomics (QTAP); *2*) quantify and compare the impact of PRHs on the concentrations of key non-CYP DMEs and transport proteins in SCHH; and *3*) evaluate the relative contribution of individual PRHs to the observed effects. These objectives were accomplished by exposing SCHH from multiple hepatocyte donors to PRHs administered individually or in combination across a range of concentrations, or known nuclear receptor activators, and then quantifying changes in absolute protein concentrations across a panel of 42 key non-CYP DMEs and transport proteins by QTAP.

## Materials and methods

### Reagents and chemicals

Reagents were obtained from Life Technologies Corporation (Carlsbad, CA, United States) unless otherwise indicated. Estrone (E1), estradiol (E2), estriol (E2), P4, CRT, rifampicin (RIF), 6-(4-chlorophenyl)imidazo[2,1-b][1,3]thiazole-5-carbaldehyde-O-(3,4-dichlorobenzyl)oxime (CITCO), chenodeoxycholic acid (CDCA), piperazine-N,N′-bis(2-ethanesulfonic acid) (PIPES), phenylmethylsulfonyl fluoride (PMSF), sucrose, and dimethyl sulfoxide (DMSO) were obtained from Sigma Aldrich (St. Louis, MO, United States). Placental growth hormone (pGH) was purchased from R&D Systems (Minneapolis, MN, United States). QualGro^™^ Seeding, QualGro^™^ Culture, and QualGro^™^ Induction media were obtained from BioIVT (Durham, NC); Matrigel Matrix and Corning Biocoat^™^ collagen I coated plates were purchased from Corning (Corning, NY, United States). Enzyme-linked immunosorbent assay (ELISA) kits for E2, E3, P4, and CRT were purchased from Cayman Chemical (Ann Arbor, MI, United States). Ethylenediaminetetraacetic acid (EDTA) and digitonin were obtained from EMD Millipore (Burlington, MA, United States).

### Sandwich-cultured human hepatocytes

Cryopreserved human primary hepatocytes were obtained from Life Technologies Corporation (Carlsbad, CA) or BioIVT (Durham, NC, United States). The hepatocytes were transporter (Hu8339, Hu8375, Hu1970, YNM) or induction (Hu8373) qualified, and all donors were adult females of reproductive age (18–49 years) as defined by the World Health Organization (Supplementary Table S1). Hepatocytes were cultured as SCHH, as previously described (Swift et al., 2010; Khatri et al., 2021b). Briefly, the cells were thawed in Hepatocyte Thaw Medium and centrifuged at 100 × *g* for 10 min. The medium was discarded, and the cell pellet was resuspended in QualGro^™^ Seeding medium and seeded on 24-well Corning Biocoat^™^ collagen I coated plates at a cell density of 250,000 cells/well. The cells were incubated at 37°C and 5% CO_2_ overnight. On day 2, the medium was replenished with QualGro^™^ Culture medium supplemented with 0.25 mg/ml Corning Matrigel^®^ Matrix, and incubated at 37°C and 5% CO_2_ for 24 h. On day 3, 4, and 5, the medium was replenished twice a day (at 9 a.m. and 5 p.m.) with QualGro^™^ Induction medium supplemented with the experimental exposure: vehicle control (0.1% DMSO), PRHs (individually or in combination as a PRH cocktail), or known activators of PXR (RIF), CAR (CITCO), or FXR (CDCA). On day 6, the cells were harvested. Cell viability was visually monitored at least once a day using a microscope over the six culture-day period*.*

### Pregnancy related hormone concentrations in hepatocyte culture medium

A dramatic increase in the synthesis and secretion of major placental-derived steroidal hormones and peptide hormones into the maternal circulation is a hallmark of pregnancy (Newbern and Freemark, 2011). In our experimental model, SCHH were exposed to the PRHs E1, E2, E3, P4, CRT, and pGH for 72 h. In the primary experiments, PRHs were administered to SCHH from each of the five donors (Hu8339, Hu8373, Hu8375, YNM, Hu1970) (Supplementary Table S1) in combination as a cocktail to mimic the simultaneous exposure to multiple PRHs that occurs during pregnancy. In secondary experiments conducted in three hepatocyte donors (Hu8373, Hu8375, and Hu1970), each PRH was administered individually to SCHH to discern their relative effects.

Due to the progressive increase in PRHs with increasing gestational age, and to elucidate the presence and magnitude of concentration-dependent effects on DME and transport protein concentration, PRH effects were evaluated across a range of physiologically relevant and supraphysiologic PRH concentrations. The average concentration of each PRH in the maternal circulation at different stages of gestation were obtained from the literature (Table 1). The targeted PRH concentration in SCHH cell culture medium in our experiments was the average concentration of each hormone in maternal plasma at different stages of gestation. In the primary experiments, four targeted PRH concentrations (T2, T3, T3-90%, and 10xT3) were investigated. Groups T2 and T3 targeted the average plasma concentration of each hormone (E1, E2, E3, P4, CRT and pGH) in the maternal circulation during trimester 2 (T2) and trimester 3 (T3), respectively. Group T3-90% targeted the upper range (90th per centile) of the T3 plasma concentration for each hormone, while group 10xT3 was a supraphysiological concentration 10-fold higher than the mean T3 concentration. In the secondary individual PRH experiments, only the T3 and 10xT3 concentrations were included.

**TABLE 1:** Circulating concentrations of key pregnancy related hormones in human pregnancy.

PRH	T2	T3	T3-90%	10xT3	Reference
E1 (nM)	18	42	70	420	Schock et al. (2016)
E2 (nM)	37	80	121	800	Schock et al. (2016)
E3 (nM)	18	33	54	330	Cohn et al. (2017)
P4 (nM)	164	424	636	4240	Schock et al. (2016)
CRT (nM)	800	800	1317	8000	Soldin et al. (2005)
pGH (nM)	0.4	1.34	3.13	13.4	Chellakooty et al. (2004)

The listed concentrations were the target concentrations (nM) in our hepatocyte model system. The T2 and T3 columns represent the reported mean plasma concentrations of each hormone in pregnant women during trimester 2 (T2) and trimester 3 (T3). The column T3-90% represents the 90th percentile (E1, E2, P4), 95th percentile (E3), 97.5th percentile (CRT), or upper range (pGH) of the T3 plasma concentrations reported in the respective studies. The column 10xT3 represents a supraphysiologic concentration that is 10-fold higher than the mean T3 concentration.

Due to rapid metabolism of estrogens and P4 in human hepatocytes, with half-lives of approximately 1–2 h, the concentrations of E1, E2, E3, and P4 exogenously administered to SCHH in our experiments needed to be increased to maintain the desired average targeted concentrations (summarized in Table 1) in the SCHH medium over the treatment period (Zhang et al., 2015). The estimated treatment concentrations for each PRH in our SCHH experimental model that would yield the desired average targeted concentrations for each PRH are summarized in Table 2. The adjusted treatment concentration was derived by simulating the projected area under the concentration-time curve (AUC) for each hormone over 24 h (AUC/τ) in SCHH using a literature-derived half-life of 2 h for estrogens (E1, E2 and E3) and 1.5 h for P4 (Zhang et al., 2015). Since CRT depletion in hepatocytes is minimal and no literature data was available on the stability of pGH in hepatocyte culture, CRT and pGH were assumed to be stable in SCHH (Lombardo et al., 2011; Zhang et al., 2015). In order to confirm the literature-derived half-life estimates, we then quantified E2, E3, P4 and CRT concentrations in SCHH medium from two qualified hepatocyte donors by ELISA per the manufacturer instructions (Cayman Chemical, Ann Arbor, MI, United States), and calculated the half-life. Results confirmed the literature reported values in our experimental model (Supplementary Table S2).

**TABLE 2:** Concentrations of exogenously administered pregnancy related hormones (PRH) in the experimental model.

PRH	T2	T3	T3-90%	10xT3	Estimated Half-life (hr)*
E1 (nM)	125	250	450	2500	2
E2 (nM)	225	500	750	5000	2
E3 (nM)	125	250	450	2500	2
P4 (nM)	1000	2500	3750	25,000	1.5
CRT (nM)	800	800	1300	8000	Stable
pGH (nM)	0.35	1.34	3.13	13.4	Stable

The listed concentrations (nM) are the concentrations of each PRH exogenously administered to the cultured human hepatocyte medium in the experimental model. Based on the elimination half-life estimates* of each PRH in human hepatocytes (summarized in Supplementary Table S2), these are the PRH treatment concentrations needed to achieve the desired average target concentration of each PRH (summarized in Table 1).

Cocktail and individual PRH experiments were carried out as previously described (Khatri et al., 2021a; Khatri et al., 2021b). Briefly, SCHH were cultured for 72 h (day-3 to day-5) in QualGro^™^ Induction medium supplemented with vehicle control, PRH (using the treatment concentrations summarized in Table 2), or nuclear receptor activators (RIF 10 μM, CITCO 1 μM, or CDCA 100 µM). The medium was replenished at 8, 16, 24, 32, 48, and 56 h after the start of treatment. At 72 h, the cells were harvested for isolation of insoluble and soluble protein. All experiments included three to four experimental replicates in each treatment group.

### Membrane-associated protein isolation

Cytosolic and membrane proteins were fractionated using detergent differential fractionation (DDF) buffer as previously described with minor modification (Qasem et al., 2021). Briefly, on day 6, the culture medium was discarded, and the cells were washed with ice-cold PBS supplemented with 1 mM PMSF. Then, 250 µl of ice-cold cytosolic extraction buffer composed of 0.015% digitonin, 10 mM PIPES, 300 nM sucrose, 100 nM NaCl, 3 mM MgCl_2_, 5 mM EDTA, and 1 M PMSF was added directly to the wells. The cells were transferred to fresh Eppendorf tubes, and incubated at 4°C for 10 min followed by centrifugation at 16,000 × *g* at 4°C for 15 min. The supernatant (i.e., cytosolic fraction) was collected and stored at −80°C. The pellet was resuspended in 150 µl of Triton X-100 extraction buffer composed of 0.5% Triton X-100, 10 mM PIPES, 300 mM sucrose, 100 nM NaCl, 3 mM MgCl_2_, 5 mM EDTA, and 1M PMSF, and incubated at 4°C for 30 min followed by a 15 min centrifugation at 16,000 × *g* at 4°C. Total protein concentration of the supernatant was determined using Bio-Rad Protein Assay Kit II (Hercules, CA, United States).

### Quantitative targeted absolute proteomics

Absolute protein concentration of a panel of 42 non-CYP phase I and phase II DMEs (Supplementary Table S3) and hepatic transport proteins (Supplementary Table S4) was quantified in the membrane fraction as previously described (Fallon et al., 2013; Khatri et al., 2019). Briefly, 20 µg of membrane fraction samples and of human liver microsome (HLM, quality control) was evaporated to dryness and resuspended in 1% sodium deoxycholate followed by denaturation and reduction. Then, the samples were digested with trypsin overnight (20 h) and the reaction was quenched with 10% trifluoracetic acid. Stable isotope labelled (SIL) peptides (0.5 or 1 pmol) with tryptic tag at the C-terminus (JPT, Berlin, Germany) were added into each sample before digestion while SIL peptides without tryptic tag at the C-terminus (Thermo Scientific Biopolymers, Rockford, IL, United States) were added after digestion. The samples were then vortexed and centrifuged at 13K × *g* for 5 min. The supernatant was collected in a fresh tube followed by phase extraction in Phenomenex Strata X 33u Polymeric Reversed Phase cartridges, and the peptides were eluted with 60% acetonitrile/40% formic acid (0.1%) and evaporated to dryness. The samples were reconstituted in 50 µl 2% acetonitrile/98% formic acid 0.1% (modified mobile phase, *see* below), centrifuged at 13K × *g* for 5 min, and the supernatant was transferred to deactivated LC-MS inserts. The samples (0.2 µl) were injected onto a nanoAcquity column: BEH130 C18 column: 150 μm × 100 mm, 1.7 μm particle size (Waters, Milford, MA, United States) coupled to a SCIEX QTRAP 5500 hybrid mass spectrometer (Framingham, MA, United States) equipped with a NanoSpray III source for chromatographic separation and subsequent MS/MS (multiple reaction monitoring [MRM] in the positive mode) analysis, respectively. The LC solvents were 0.1% formic acid/acetonitrile (99:1) and 100% acetonitrile, and a gradient elution was achieved at a flow rate of 1.3 μl/min as described in (Fallon et al., 2013). For each DME and transport protein studied, 1-3 SIL peptide standards were used; Supplementary Tables S3, S4 present the SIL peptides used to report absolute concentration of each DME and transport protein, respectively.

The nanoLC-MS/MS chromatograms were visualized and peak area integration was performed using Skyline 21.1 software (Pino et al., 2020). The LC-MS peak for the SIL standard was used to quantify the absolute concentration of the corresponding protein. The absolute concentration of each protein was determined using the peak area ratio of analyte to standard peptide, normalized to the protein content of the sample*,* and reported the concentration as pmol per mg protein. A lower limit of quantitation (LLOQ) of 0.1 pmol/mg was applied to all proteins in all samples. If the mean concentration of a specific protein in the vehicle control group was less than 0.1 pmol/mg (i.e., LLOQ), the protein was considered unquantifiable in that hepatocyte donor. Proteins that were unquantifiable in one or two hepatocyte donors included OAT2 (YNM), MATE1 (YNM and Hu1970), OSTα (Hu8373 and YNM), NTCP (Hu8339 and YNM), and BCRP (Hu8339 and Hu8373). Proteins that were unquantifiable in three or more donors (UGT1A7, UGT1A8, UGT1A10, UGT2B17 [due to possible genetic polymorphism (Fallon et al., 2013)], OATP1A2, ENT2, OSTβ, MRP4, and MRP6) were not considered in the induction experiment analysis.

### Data analysis

Data are presented as mean ± SEM and expressed as an absolute protein concentration or as a fold-change relative to vehicle control, unless otherwise indicated. Data analysis was first conducted across biological replicates (*n* = 3–4 per experimental group) within each hepatocyte donor. Within each donor, the absolute concentration of a given protein in each sample was divided by the mean concentration in the control group to calculate the fold-change of each protein in each experimental group relative to vehicle control. Then, the mean fold-change data within each hepatocyte donor for each treatment group was carried forward as a single data point into an analysis of the net effect across the five hepatocyte donors. For proteins with unquantifiable concentrations in one or two select donors, only data from the hepatocyte donors with quantifiable basal protein concentration (>0.1 pmol/mg in the vehicle control group) were considered in the across-donor induction analysis. All data were log-normalized prior to statistical analysis. Comparisons across experimental groups was performed using a one-way ANOVA (α = 0.05) with a post-hoc Fisher’s LSD test to evaluate differences across each group. Statistical significance was defined as *p* < 0.05. The data analysis was carried out with GraphPad Prism 9.1 (GraphPad Software, La Jolla, CA, United States) and Microsoft Excel (Microsoft, WA, United States).

## Results

### Quantification of multiple DMEs and transport proteins in SCHH

**FIGURE 1:**
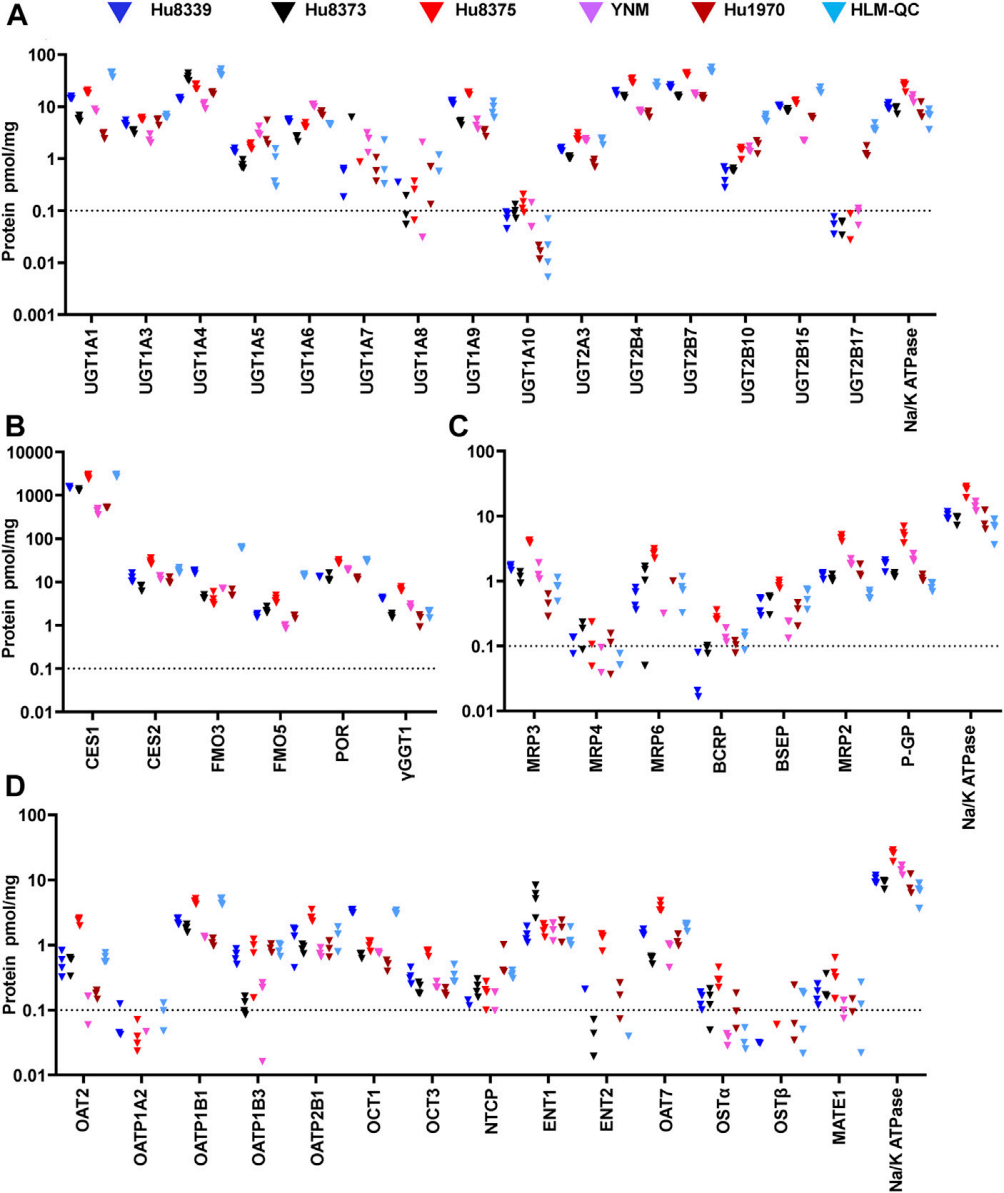
Basal absolute protein concentrations of DMEs and transport proteins in SCHH. Absolute concentrations of 15 UGTs **(A)**, six other DMEs **(B)**, seven ABC transport proteins **(C)**, and 14 SLC transport proteins **(D)** in SCHH. Concentrations of each DME and transport protein, and Na^+^/K ATPase, were quantified in membrane fractions isolated from five qualified hepatocyte donors under basal conditions and in a human liver microsome quality control (HLM-QC) sample. The SCHH samples included three to four biological replicates in each donor, and the HLM-QC sample was analyzed in duplicate. The dotted line at y = 0.1 pmol/mg represents lower limits of quantitation (LLOQ).

We used an isotope dilution quantitative proteomic method (Fallon et al., 2013; Khatri et al., 2019) to quantify the basal absolute protein concentration of 42 non-CYP DMEs and drug transport proteins in SCHH membrane fractions from five female hepatocyte donors. The proteins were categorized into four groups: UGTs (15 proteins), other DMEs (six proteins), SLC transport proteins (14 proteins), and ABC transport proteins (seven proteins) (Figures 1A,B). Proteins with a mean basal concentration >0.1 pmol/mg across all five donors and >0.1 pmol/mg within at least three of the five hepatocyte donors were considered quantifiable. Of the total 42 proteins, 33 (78.6%) were quantifiable; this included 28 (66.7%) proteins that were quantifiable in all five donors and an additional 5 (11.9%) proteins that were quantifiable in at least three donors (OAT2, MATE, OSTα, NTCP, and BCRP). In contrast, 9 (21.4%) proteins were unquantifiable in three or more donors (UGT1A7, UGT1A8, UGT1A10, UGT2B17, OATP1A2, ENT2, OSTβ, MRP4, and MRP6) and thus were not considered in the induction experiments.

### PXR, CAR and FXR activators alter concentrations of certain DMEs and transport proteins in SCHH

**FIGURE 2:**
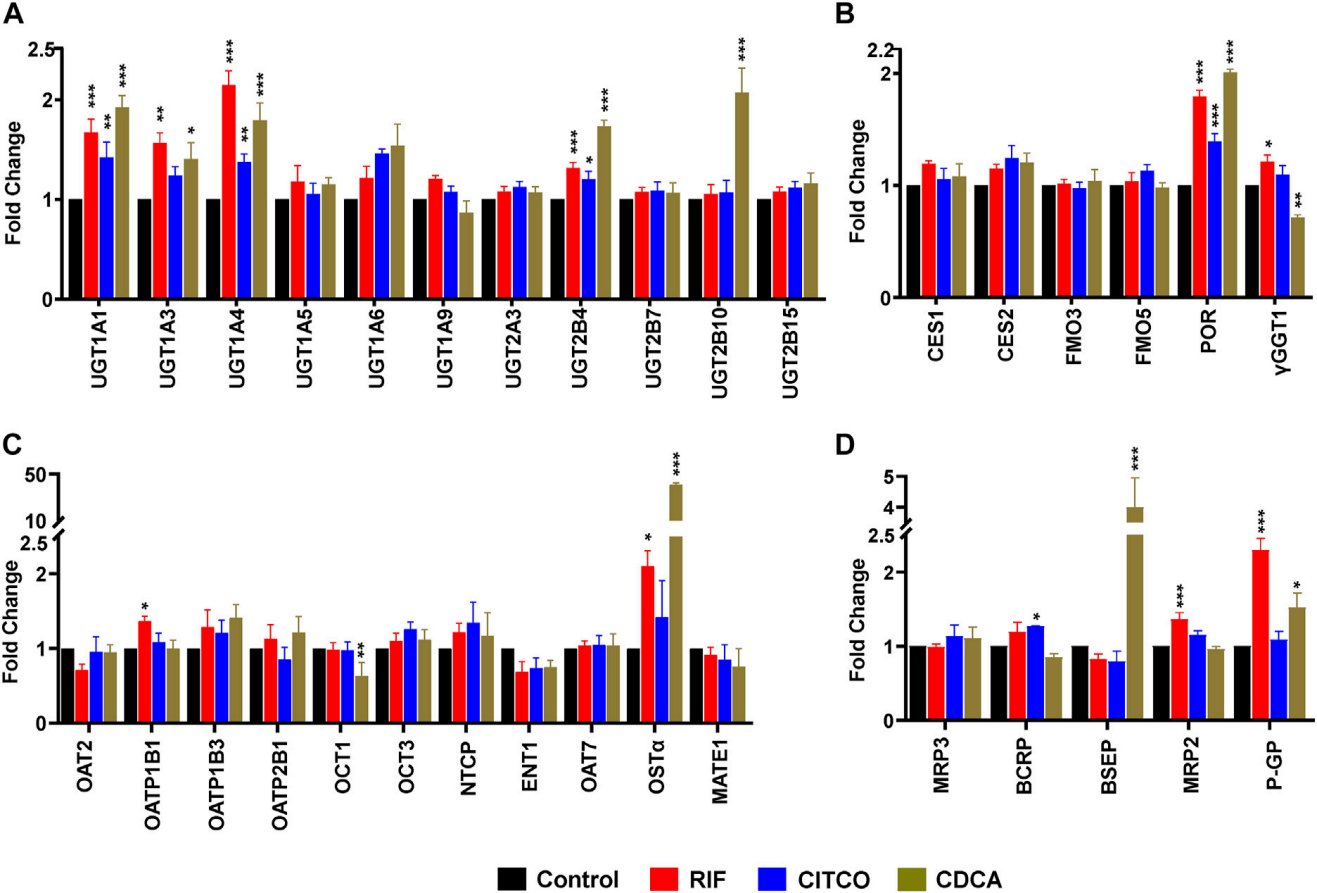
Nuclear receptor activator induced alterations in DME and transport protein concentration in SCHH. Absolute protein concentration of 11 UGTs **(A)**, six other DMEs **(B)**, 11 SLC transport proteins **(C)**, and five ABC transport proteins **(D)** were quantified in SCHH from five qualified in hepatocyte donors following exposure to vehicle control, rifampicin (RIF, 10 µM), CITCO (1 µM), or chenodeoxycholic acid (CDCA, 100 µM) for 72 h. The experiment in each hepatocyte donor included three to four biological replicates, and mean fold-change was computed for each protein relative to the vehicle control group within each donor. The data reflect the mean ± SEM fold-change for each protein relative to control across donors (*n* = 5 per group). *p < 0.05, **p < 0.01, ***p < 0.001 versus control.

To test the dynamic sensitivity of our experimental SCHH system, we quantified changes in concentrations of 11 UGT (Figure 2A), six other DMEs (Figure 2B), 10 SLC transport proteins (Figure 2C), and six ABC transport proteins (Figure 2D) in SCHH membrane fractions following exposure to prototypical nuclear receptor activators of PXR (rifampicin), CAR (CITCO), and FXR (CDCA). Rifampicin significantly increased concentrations of four UGT (UGT1A1, UGT1A3, UGT1A4, and UGT2B4), two other DMEs (POR and γGGT1), and four transport proteins (OATP1B1, OSTα, MRP2, and P-GP) across donors. CITCO significantly increased concentration of four DMEs (UGT1A1, UGT1A4, UGT2B4, POR) and one transport protein (BCRP). CDCA significantly increased protein concentration of five UGT (UGT1A1, UGT1A3, UGT1A4, UGT2B4, UGT2B10), one other DME (POR), and three transport proteins (OSTα, BSEP, and P-GP). The CDCA evoked increases in OSTα (40.7 ± 1.8-fold) and BSEP (4.0 ± 1.0-fold) protein concentrations were the largest observed effects. While rifampin and CITCO did not suppress expression of any target proteins, CDCA also significantly decreased the concentration of two proteins (γGGT1 and OCT1). None of the three ligands significantly altered concentration of CES or FMO proteins.

### PRHs alter concentrations of multiple DME and transport proteins in an isoform-specific manner

**FIGURE 3:**
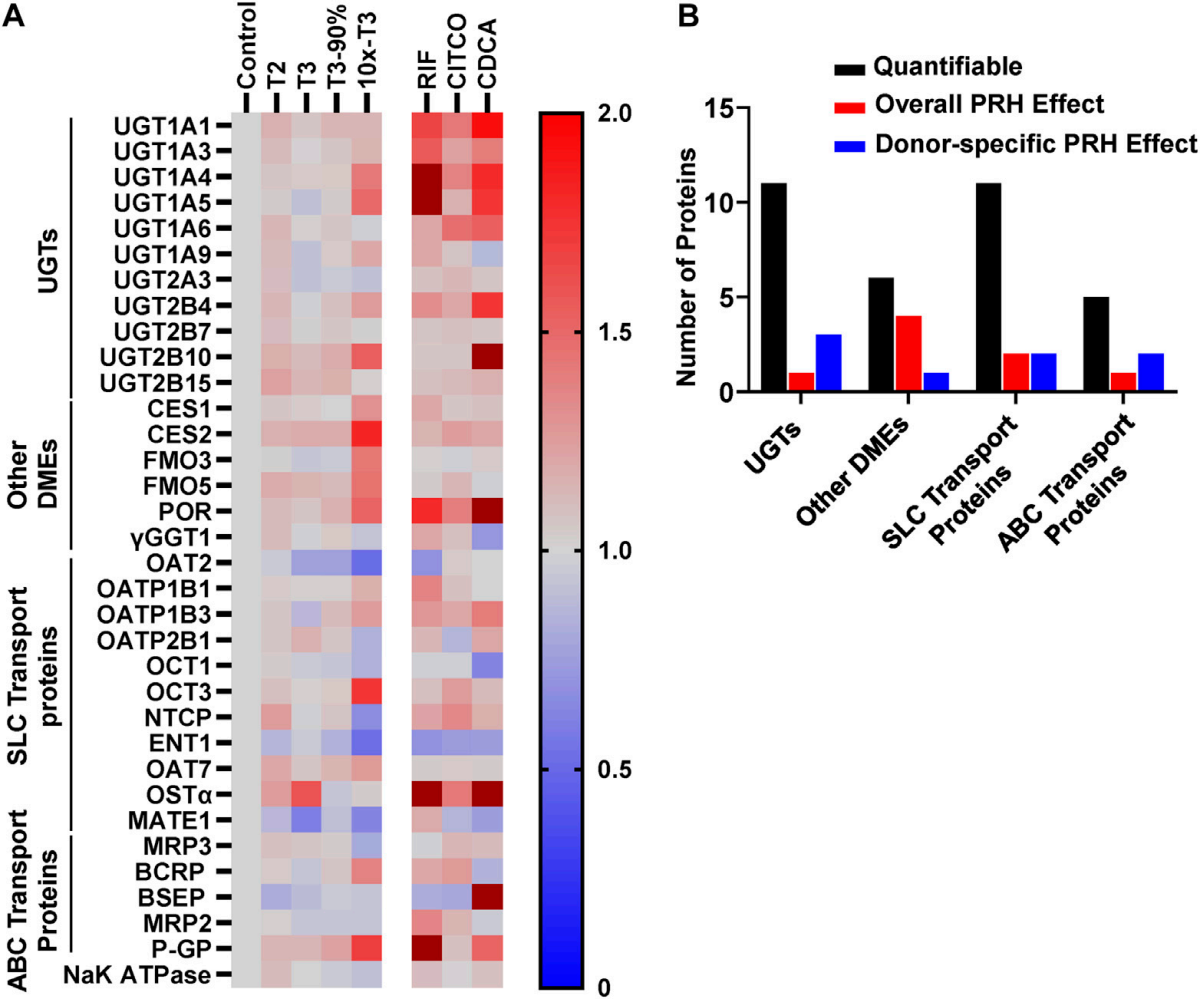
PRHs altered the absolute protein concentration of DMEs and transport proteins in SCHH in an isoform and concentration dependent manner. SCHH from five qualified donors were exposed to vehicle control, PRH cocktails, or nuclear receptor activators. The PRH cocktails target average trimester 2 (T2), average trimester 3 (T3), upper range of T3 (T3-90%), and supraphysiological (10xT3) PRH concentrations (Table 1). The experiment in each hepatocyte donor included three to four biological replicates, and mean fold-change was calculated for each protein relative to the vehicle control group within each donor. **(A)** Heat map summarizing the mean increase, no change, or decrease in concentrations of 33 DMEs and transport proteins across donors relative to the vehicle control group. The impact of RIF, CITCO, and CDCA are presented on the right side of the heat map for comparative purposes. *Color index*: Deep red (fc > 2) Red (fc > 1), Gray, (fc = 1), and blue (fc < 1). **(B)** Summary of the number of quantifiable proteins (by protein type) that exhibited a significant net alteration by PRH (ANOVA *p* < 0.05 for overall fold-change across all five donors) or exhibited a donor-specific alteration by PRH (ANOVA *p* < 0.05 within two or more donors, but ANOVA *p* > 0.05 for overall fold-change across all five donors).

Absolute concentrations of 33 DMEs and transport proteins were quantified in SCHH exogenously exposed to PRH cocktails that target average T2, average T3, the upper range of T3 (T3-90%), and supraphysiological (10xT3) PRH concentrations. A heatmap illustrating the average impact of PRH on DME and transport protein concentrations, relative to vehicle control, across donors revealed isoform-specific and concentration-dependent effects (Figure 3A). The corresponding average fold-change of each protein in each PRH treatment group is summarized in Supplementary Table S5.

Overall, as summarized in Figure 3B, eight of 33 (24%) proteins (UGT1A4, CES1, CES2, FMO5, POR, OAT2, OCT3, and P-GP) exhibited a significant PRH-evoked net change in protein concentration relative to vehicle control (ANOVA *p* < 0.05) across five hepatocyte donors. An additional eight (24%) proteins (UGT1A1, UGT2B4, UGT2B10, FMO3, OCT1, ENT1, MRP2, MRP3) exhibited significant PRH alterations within at least two individual donors (ANOVA *p* < 0.05), but the observed net PRH effect across all five donors was not significant (ANOVA *p* > 0.05). Together, four of 11 UGTs (36%), five of six other DMEs (83%), four of 11 SLC transport proteins (36%), and three of five ABC transport proteins (60%) exhibited an overall or donor specific PRH effect. In contrast, the majority of proteins (17 of 33, 52%) exhibited no discernable impact by PRHs either within or across hepatocyte donors.

### PRHs increase UGT protein concentrations in SCHH in an isoform-specific manner

**FIGURE 4:**
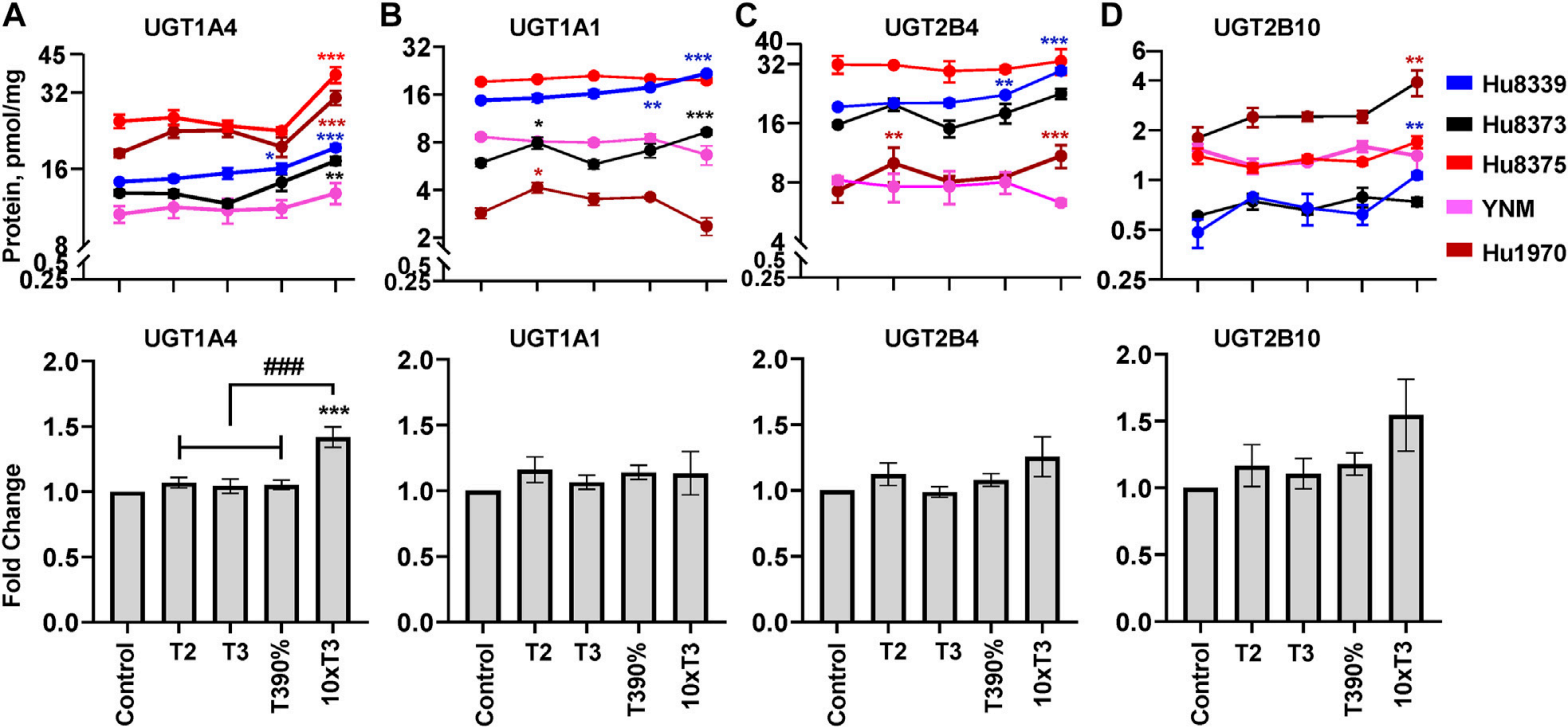
PRHs increased the absolute protein concentration of key UGT isoforms in SCHH. Human primary hepatocytes from qualified donors were exposed to vehicle control or PRH cocktails. The PRH cocktails target average trimester 2 (T2), average trimester 3 (T3), upper range of T3 (T3-90%), and supraphysiological (10xT3) PRH concentrations (Table 1). The line graphs represent mean ± SEM absolute protein concentration of UGT1A4 **(A)**, UGT1A1 **(B)**, UGT2B4 **(C)**, and UGT2B10 **(D)** in SCHH in response to exposure to PRHs within each hepatocyte donor (*n* = 3–4 biological replicates per group). The bar graphs below represent mean ± SEM fold-change for each UGT relative to control across all donors (*n* = 5 per group). *p < 0.05, **p < 0.01, ***p < 0.001 versus control. #Represents the concentration-dependent effect across PRH groups (###*p* < 0.001).

The impact of PRHs on absolute protein concentrations of the 11 quantifiable UGT isoforms within each hepatocyte donor and the net effect across hepatocyte donors was evaluated. Overall, four UGT proteins exhibited either a significant net PRH effect across all five donors (UGT1A4) or PRH effects that were significant within two or more individual donors (UGT1A1, UGT2B4, UGT2B10) (Figure 4). The remaining seven UGT isoforms (UGT1A3, UGT1A5, UGT1A6, UGT1A9, UGT2A3, UGT2B7, UGT2B15) did not exhibit altered protein concentration in SCHH in response to PRH exposure (Supplementary Figure S1).

Evaluation of the average net effect across donors demonstrated that PRHs significantly increased UGT1A4 protein concentrations in SCHH relative to vehicle control (ANOVA *p* < 0.001) (Figure 4A). The PRH induced increase of UGT1A4 protein concentration was not evident at the T2, T3 and T3-90% concentrations, and was only observed at the 10xT3 concentration. At this supraphysiologic PRH concentration, UGT1A4 protein concentration increased by 1.42 ± 0.08-fold compared to the vehicle control (*p* < 0.001), and these significant effects were observed within four of five hepatocyte donors (Figure 4A).

Although PRHs did not evoke a significant net change in UGT1A1, UGT2B4, and UGT2B10 protein concentrations across donors (ANOVA *p* > 0.05), PRHs significantly increased protein concentrations of these UGT isoforms within two donors (Figures 4B–D). PRH increased UGT1A1 in donors Hu8339 (ANOVA *p* < 0.001) and Hu8373 (ANOVA *p* = 0.002) (Figure 4B), UGT2B4 in donors Hu8339 (ANOVA *p* < 0.001) and Hu1970 (ANOVA *p* = 0.006) (Figure 4C), and UGT2B10 in donors Hu8339 (ANOVA *p* = 0.012) and Hu1970 (ANOVA *p* = 0.028) (Figure 4D). Similar to UGT1A4, PRH increased UGT1A1 (1.48 ± 0.05-fold in Hu8339 and 1.56 ± 0.07-fold in Hu8373), UGT2B4 (1.53 ± 0.05-fold in Hu8339 and 1.44 ± 0.09-fold in Hu1970), and UGT2B10 (2.21 ± 0.10-fold in Hu8339 and 1.22 ± 0.07-fold in Hu1970) at the 10xT3 concentration.

### PRHs increase other DME protein concentrations in SCHH in an isoform-specific manner

**FIGURE 5:**
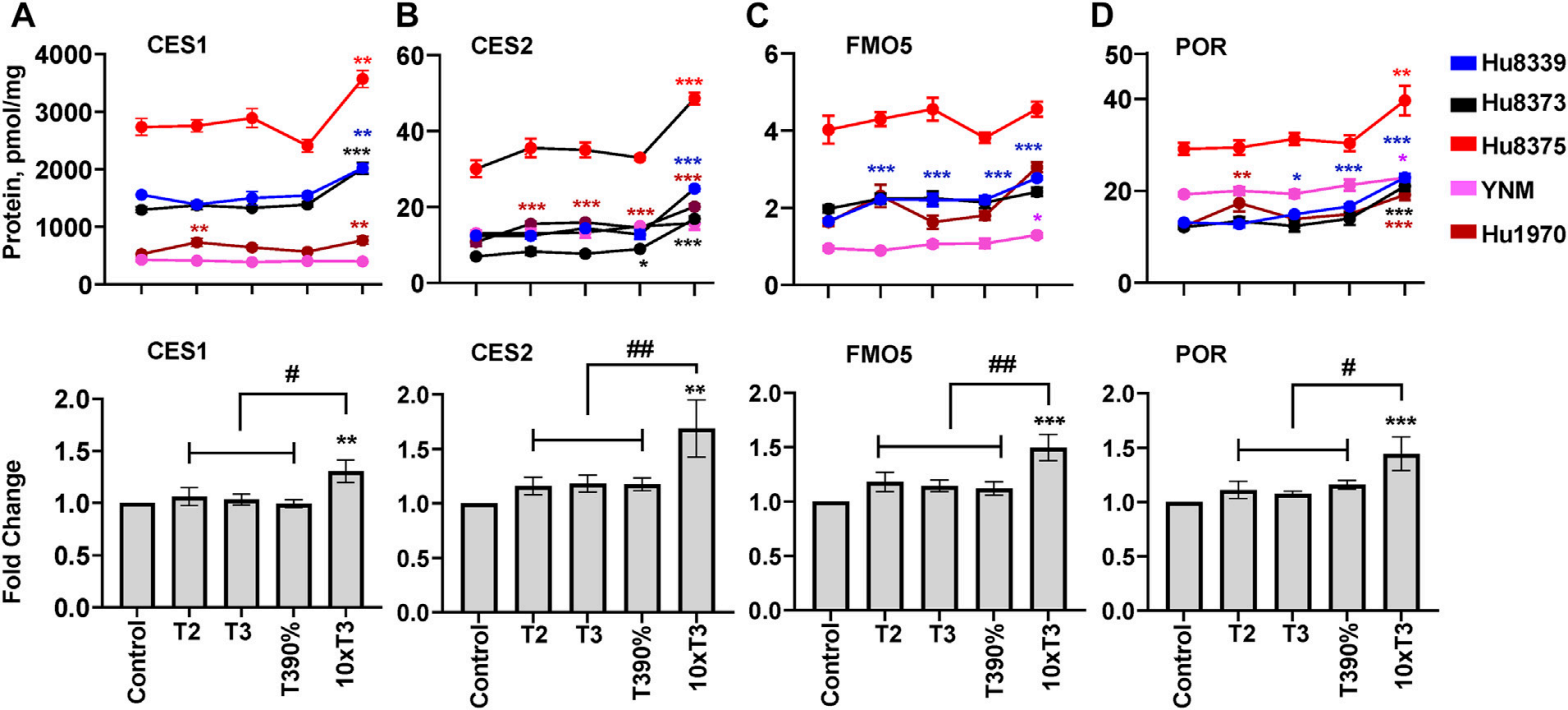
PRHs increased the absolute protein concentration of other key DMEs in SCHH. Human primary hepatocytes from qualified donors were exposed to vehicle control or PRH cocktails. The PRH cocktails target average trimester 2 (T2), average trimester 3 (T3), upper range of T3 (T3-90%), and supraphysiological (10xT3) PRH concentrations (Table 1). The line graphs represent mean ± SEM absolute protein concentration of CES1 **(A)**, CES2 **(B)**, FMO5 **(C)**, and POR **(D)** in SCHH in response to exposure to PRHs within each hepatocyte donor (*n* = 3–4 biological replicates per group). The bar graphs below represent mean ± SEM fold-change for each protein relative to control across all donors (*n* = 5 per group). *p < 0.05, **p < 0.01, ***p < 0.001 versus control. #Represents concentration-dependent effect across PRH groups (# < 0.05, ## < 0.01).

The impact of PRHs on absolute protein concentrations of six other key non-CYP metabolism proteins within each hepatocyte donor and the net effect across hepatocyte donors was evaluated. Evaluation of the average net PRH effect across donors demonstrated a significant increase in absolute protein concentrations of CES1 (ANOVA *p* = 0.032; Figure 5A), CES2 (ANOVA *p* = 0.017; Figure 5B), FMO5 (ANOVA *p* = 0.002; Figure 5C), and POR (ANOVA *p* = 0.012; Figure 5D) relative to vehicle control. A significant PRH mediated increase of these proteins was not observed at the T2, T3, or T3-90% concentrations. However, compared to vehicle control, exposure to the 10xT3 concentration significantly increased CES1 (1.31 ± 0.11-fold, *p* = 0.007), CES2 (1.81 ± 0.20-fold, *p* = 0.001), FMO5 (1.45 ± 0.14-fold, *p* < 0.001) and POR (1.52 ± 0.11-fold, *p* = 0.001) protein concentrations across donors. At this supraphysiological PRH concentration, significant effects were observed within four of five of hepatocyte donors for CES1 and CES2, two of five donors for FMO5, and all five donors for POR (Figures 5A–D).

Although PRHs did not induce a significant net change in FMO3 protein concentrations across all donors (ANOVA *p* = 0.104), a significant increase in FMO3 expression was observed within four of the five donors (Supplementary Figure S2A): Hu8339 (ANOVA *p* = 0.008), Hu8375 (ANOVA *p* = 0.010), YNM (ANOVA *p* = 0.035), and Hu1970 (ANOVA *p* < 0.001). The effects were only evident at the 10xT3 concentration, which increased FMO3 protein concentrations by 1.17 ± 0.027-fold in donor Hu8339, 1.60 ± 0.12-fold in donor Hu8375, 1.21 ± 0.12-fold in donor YNM, and 1.95 ± 0.09-fold in donor Hu1970, respectively. In contrast, PRHs did not impact γGGT1 across donors (ANOVA = 0.442), and an unpredictable pattern of PRH effects was observed within individual donors (Supplementary Figure S2B).

### PRHs alter transport protein concentration in SCHH in an isoform-specific manner

**FIGURE 6:**
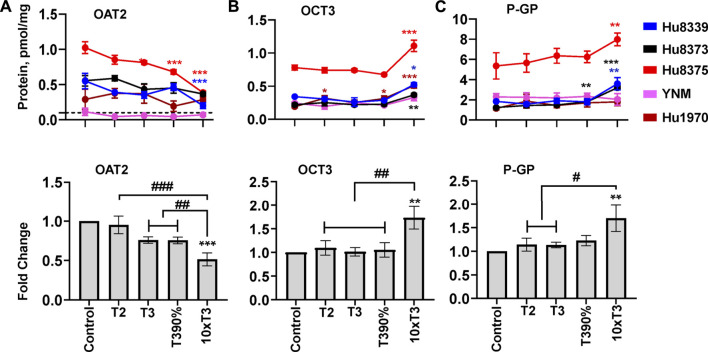
PRHs altered the absolute protein concentration of key transport proteins in SCHH. Human primary hepatocytes from qualified donors were exposed to vehicle control or PRH cocktails. The PRH cocktails target average trimester 2 (T2), average trimester 3 (T3), upper range of T3 (T3-90%), and supraphysiological (10xT3) PRH concentrations (Table 1). The line graphs represent mean ± SEM absolute protein concentration of OAT2 **(A)**, OCT3 **(B)**, and P-GP **(C)** in SCHH in response to exposure to PRHs within each hepatocyte donor (*n* = 3–4 biological replicates per group). The bar graphs below represent mean ± SEM fold-change for each protein relative to control across all donors (*n* = 4–5 per group). The dotted line at y = 0.1 pmol/mg represents lower limits of quantitation. OAT2 was not quantifiable in donor YNM, and thus the fold-change data for OAT2 included *n* = 4 per group. *p < 0.05, **p < 0.01, ***p < 0.001 versus control. #Represents concentration-dependent effect across PRH groups (# < 0.05, ## < 0.01, ### < 0.001).

The impact of PRHs on absolute protein concentrations of the 16 quantifiable SLC and ABC transport proteins within each hepatocyte donor and the net effect across all hepatocyte donors was evaluated. Overall, three transport proteins (OAT2, OCT3, and P-GP) showed a significant (ANOVA *p* < 0.05) net effect in response to PRH across donors (Figure 6), while an additional two SLC transport proteins (OCT1 and ENT1; Supplementary Figures S3A,B) and two ABC transport proteins (MRP2 and MRP3; Supplementary Figures S4A,B) exhibited PRH effects that were significant within two or more individual donors. The remaining seven SLC transport proteins (OATP1B1, OATP1B3, OATP2B1, NTCP, OSTα, OAT7, MATE1) (Supplementary Figures S3C–I) and two ABC transport proteins (BCRP, BSEP) (Supplementary Figures S4C,D) did not exhibit altered protein concentrations in response to PRH exposure across or within donors.

Evaluation of the average net effect across donors relative to vehicle control demonstrated that PRHs significantly decreased OAT2 (ANOVA *p* = 0.001; Figure 6A), increased OCT3 (ANOVA *p* = 0.008; Figure 6B), and increased P-GP (ANOVA *p* = 0.045; Figure 6C) protein concentrations. The observed decrease in OAT2 protein concentration was evident, but not statistically significant, at the T3 (0.76 ± 0.04-fold, *p* = 0.063) and T3-90% (0.76 ± 0.04-fold, *p* = 0.060) concentrations; a significant decrease was observed at the 10xT3 concentration (0.51 ± 0.08-fold, *p* = 0.001) (Figure 6A). For OCT3 and P-GP, an increase was not observed at the T2, T3, and T3-90% concentrations compared to vehicle control; however, the 10xT3 PRH concentration significantly increased OCT3 (1.73 ± 0.24-fold, *p* = 0.002) and P-GP (1.70 ± 0.28-fold, *p* = 0.004) protein concentrations. At this supraphysiological PRH concentration, significant changes compared to control were observed within four of five hepatocyte donors for OCT3 and three of five donors for P-GP (Figures 6B,C).

Although PRHs did not evoke a significant net change in OCT1, ENT1, MRP2, and MRP3 protein concentrations across donors (ANOVA *p* > 0.05), PRHs significantly decreased these transport proteins within two or more hepatocyte donors (Figure 6). PRH significantly decreased OCT1 in donors Hu8375 (ANOVA *p* = 0.004) and YNM (ANOVA *p* = 0.004) (Supplementary Figure S3A), ENT1 in donors Hu8339 (ANOVA *p* = 0.010), Hu8373 (ANOVA *p* = 0.006) and Hu8375 (ANOVA *p* = 0.002) (Supplementary Figure S3B), MRP2 in donors Hu8375 (ANOVA *p* < 0.001) and YNM (ANOVA *p* < 0.001) (Supplementary Figure S4A), and MRP3 in donors Hu8375 (ANOVA *p* < 0.001) and YNM (ANOVA *p* = 0.002) (Supplementary Figure S4B). These alterations were observed mainly at the supraphysiological 10xT3 concentration (OCT1: 0.64 ± 0.05-fold in Hu8375 and 0.52 ± 0.03-fold in YNM; ENT1: 0.58 ± 0.08-fold in Hu8339, 0.22 ± 0.02-fold in Hu8373, and 0.69 ± 0.01-fold in Hu8375; MRP2: 0.645 ± 0.035-fold in Hu8375 and 0.59 ± 0.035-fold in YNM; MRP3: by 0.65 ± 0.02-fold in Hu8375 and 0.43 ± 0.03-fold in YNM).

### Contribution of individual hormones

**FIGURE 7:**
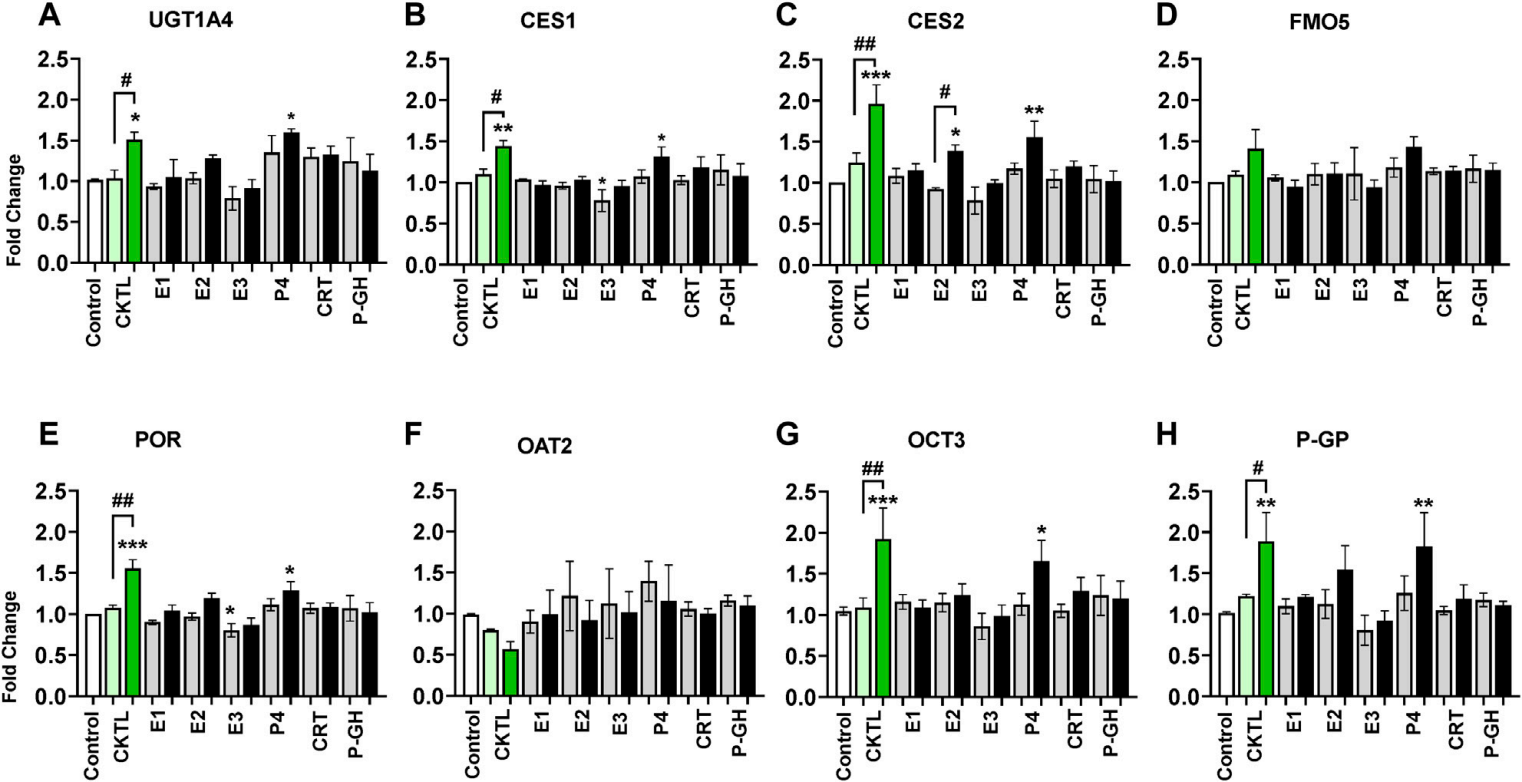
Impact of individual pregnancy related hormones (iPRHs) on DME and transport protein concentrations in SCHH. Human hepatocytes from three qualified donors (Hu8373, Hu8375, and Hu1970) were exposed to vehicle control, PRH cocktails (CKTL), or the iPRHs E1, E2, E3, P4, CRT, or pGH that target average trimester 3 (transparent bar) or supraphysiological 10-fold T3 (solid bar) PRH concentrations. The bar graphs represent mean ± SEM fold-change for UGT1A4 **(A)**, CES1 **(B)**, CES2 **(C)**, FMO5 **(D)**, POR **(E)**, OAT2 **(F)**, OCT3 **(G)**, and P-GP **(H)** (proteins that were significantly altered by PRH cocktails in Figures 4–6) relative to control across donors in response to the PRH CKTL or iPRHs (*n* = 3 per group). *p* < 0.05, **p < 0.01, ***p < 0.001 versus control. #Represents concentration-dependent effect across PRH groups (# < 0.05, ## < 0.01).

To evaluate the relative contribution of individual PRHs to the observed effects of the PRH cocktails, we exposed SCHH from three donors to individual PRHs (E1, E2, E3, P4, P4, CRT, and pGH). Among the eight proteins significantly altered by the PRH cocktails across hepatocyte donors, six proteins (UGT1A4, CES1, CES2, POR, OCT3, P-GP) were significantly altered by one or more individual PRHs (Figure 7). In contrast, FMO5 (Figure 7D) and OAT2 (Figure 7F) protein concentrations were not significantly altered by any of the individual PRHs.

Exposure to P4 significantly increased UGT1A4 (Figure 7A), CES1 (Figure 7B), CES2 (Figure 7C), POR (Figure 7E), OCT3 (Figure 7G), and P-GP (Figure 7H) concentrations, and E2 significantly increased CES2 concentrations. These effects were concentration-dependent and only observed at the 10xT3 concentration. E2 also appeared to modestly increase UGT1A4, POR, and P-GP concentrations, but these differences were not statistically significant. In contrast, E3 appeared to decrease concentrations of multiple proteins; although the observed differences were modest in magnitude and statistically significant for only POR. E1, CRT, and PGH did not significantly alter concentrations of these DMEs or transport proteins in SCHH.

## Discussion

Accumulating evidence has suggested that increased PRH secretion during pregnancy is a central mediator of pregnancy-associated changes in the pharmacokinetics and hepatic clearance of several drugs *via* altered hepatic DME expression (Jeong, 2010; Isoherranen and Thummel, 2013; Tasnif et al., 2016; Mulrenin et al., 2021). Previous *in vitro* studies have shown that PRHs alter the expression and function of multiple CYPs in primary human hepatocytes in an isoform-specific and concentration-dependent manner (Choi et al., 2013; Dickmann and Isoherranen, 2013; Papageorgiou et al., 2013; Zhang et al., 2015; Khatri et al., 2021b). Physiologically-based pharmacokinetic (PBPK) modeling studies have established strong correlations between PRH associated changes in CYP expression and metabolism *in vitro* with gestational changes in the clearance of prototypical probe or clinically relevant substrates of various CYPs including CYP3A4 and CYP2B6 (Dallmann et al., 2017; Dallmann et al., 2018a). However, the impact of PRHs on the expression of key non-CYP phase I DMEs, phase II DMEs, and hepatic SLC and ABC transport proteins in primary human hepatocytes remain largely unknown.

In the current study, we exposed SCHH from multiple hepatocyte donors to PRHs across a range of concentrations, and then quantified and compared the PRH evoked alterations in the absolute protein concentration of 33 non-CYP DMEs and transport proteins using QTAP. Our data demonstrated that *1*) eight of 33 (24%) proteins (UGT1A4, CES1, CES2, FMO5, POR, OAT2, OCT3, P-GP) exhibited a significant PRH-mediated net change in protein concentration across donors; *2*) an additional eight (24%) proteins (UGT1A1, UGT2B4, UGT2B10, FMO3, OCT1, ENT1, MRP2, MRP3) were significantly altered by PRH within at least two donors; *3*) the PRH effects were concentration-dependent and mostly evident following exposure to the supraphysiologic 10xT3 PRH concentration. Collectively, these findings demonstrate that PRHs alter the expression of various non-CYP DMEs and transport proteins in SCHH in a concentration-dependent and isoform-specific manner, illustrate that the presence and magnitude of PRH effects vary substantially by hepatocyte donor for certain proteins, and provide mechanistic insight into experimental and clinical studies investigating altered disposition of clinically relevant drug substrates of these proteins during pregnancy. Moreover, in the absence of DME and transporter protein concentrations in liver tissue collected from pregnant and non-pregnant individuals, data from this study also could be used to inform pharmacokinetic models of drug disposition changes in pregnancy.

Khatri *et al.* previously reported that PRHs increased mRNA levels and absolute protein concentrations of UGT1A1 and UGT1A4, but did not significantly alter UGT1A3, UGT1A6, UGT1A9 and UGT2B7 expression, in SCHH from three donors (Khatri et al., 2021a). The current experiments extend these observations by evaluating a larger panel of UGT1A and UGT2B proteins across a larger number of hepatocyte donors. Of the 11 UGTs quantified, PRHs increased UGT1A1, UGT1A4, UGT2B4, and UGT2B10 in two or more hepatocyte donors. However, the PRH mediated effects were most pronounced with UGT1A4, which was the only UGT isoform that exhibited a significant net increase in protein concentration across hepatocyte donors. Although these effects were only evident at the supraphysiologic 10xT3 concentration, induction of UGT1A4 protein in our experimental model is consistent with prior *in vitro* studies in HepG2 cells reporting E2 mediated increases in *UGT1A4* mRNA expression and lamotrigine glucuronidation *via* activation of ERα receptor (Chen et al., 2009) and prior human studies showing that lamotrigine glucuronidation, oral clearance, and dose adjustments are increased during pregnancy (Ohman et al., 2008a; Ohman et al., 2008b; Pennell et al., 2020). Together, these results suggest increased secretion of PRHs and subsequent induction of hepatic UGT1A4 expression may in part explain the increased glucuronidation and clearance of the antiepileptic drug lamotrigine in pregnant individuals.

Our observations that PRHs increased UGT1A1 protein concentration in SCHH in a concentration-dependent and hepatocyte donor-specific manner were also consistent with our prior study, which demonstrated that PRHs increase UGT1A1 expression and UGT1A1-mediated labetalol glucuronidation in SCHH, and that the magnitude of these effects vary across hepatocyte donors (Khatri et al., 2021a). These results provide further mechanistic insight into clinically observed gestational age dependent increases in labetalol oral clearance, which vary in magnitude and exhibit and inter-individual differences, in hypertensive pregnant patients (Fischer et al., 2014; Khatri et al., 2021a; Mulrenin et al., 2021). Although the functional implications remain unclear, we also report for the first time hepatocyte donor-specific increases in UGT2B4 and UGT2B10 protein concentrations in SCHH. Further studies that quantify and compare PRH effects on the hepatic glucuronidation of UGT1A1, 1A4, 2B4, and 2B10 substrates, and elucidate the underlying mechanisms are warranted.

CES1 and CES2 play a pivotal role in hepatic disposition of multiple drugs including bioactivation of prodrugs such as oseltamivir (Shi et al., 2006). We observed that PRHs increased the absolute protein concentration of CES1 and CES2 in SCHH. These effects were only observed at the supraphysiologic 10xT3 concentration and predominantly driven by P4 and E2. In contrast, a prior study in human and rodent hepatocytes suggested that E2 decreased CES1 and CES2 mRNA and protein expression (Wu et al., 2018). Moreover, hepatic *Ces1 and Ces2* mRNA levels decreased in pregnant compared to nonpregnant mice (Fortin et al., 2013), and exogenous administration of E2 (1 µg/day) to pregnant rats from gestation day 3 to day 20 decreased CES activity (Mathews and Devi, 1994). In humans, pregnancy did not alter systemic exposure to oseltamivir; although, plasma levels of oseltamivir carboxylate were lower in pregnant individuals compared to nonpregnant controls (Beigi et al., 2011). Because oseltamivir carboxylate is primarily excreted *via* the kidney, lower active metabolite exposure in pregnant individuals may be related increased renal elimination and not decreased CES1 activity (Beigi et al., 2011; Tasnif et al., 2016; Jogiraju et al., 2017). Therefore, there does not appear to be evidence supporting a CES1-mediated increase in oseltamivir carboxylation in humans. Thus, the functional and clinical relevance of increased CES expression in human hepatocytes following PRH exposure warrants further investigation.

The FMOs and cytochrome P450 reductase (POR) are also important proteins in the oxidative metabolism of drugs. POR is also integral to physiologically relevant CYP-dependent redox reactions that regulate cholesterol and bile acid homeostasis (Heintze et al., 2021). In this study, we report that PRHs increased FMO5 and POR protein concentration across hepatocyte donors and yielded a donor-specific increase in FMO3 expression. A previous study in rodent hepatocytes showed that while dexamethasone and P4 did not alter *Fmo3* mRNA levels, E2 suppressed *Fmo3* expression (Coecke et al., 1998; Esposito et al., 2014). Although the impact of pregnancy or PRHs on POR has not been studied to date, our observation that PRHs and nuclear receptor activators increased POR in SCHH was consistent with prior reports that GR and PXR activators induced *Por* mRNA levels in a rat hepatoma cell line (Riddick and Mullen Grey, 2020). However, the molecular mechanisms of POR transcriptional regulation and the functional relevance of PRH-mediated increases in POR protein concentration on drug metabolism in SCHH requires further study.

In addition to DMEs, hepatic transport proteins play an essential role in drug disposition (Giacomini et al., 2010) and are prone to altered expression in different disease states (Thakkar et al., 2017; Bezençon et al., 2019). However, the impact of pregnancy and PRHs on the expression and function of key SLC and ABC transport proteins in human hepatocytes remain largely unknown. In our SCHH model, we observed that OAT2 was significantly decreased and OCT3 and P-GP were significantly increased in response to PRH exposure. OAT2 is expressed on the basolateral membrane and drives the uptake of anionic endogenous molecules including cyclic guanosine monophosphate and clinically important drugs (Kimoto et al., 2018). Because most OAT2 drug substrates such as warfarin, sulfamethoxazole, and irinotecan (Marada et al., 2015; Kimoto et al., 2018) are contraindicated in pregnancy, the clinical significance of altered hepatic OAT2 protein concentration in pregnancy is unclear and warrants further investigation as additional OAT2 substrates such as niacin are identified (Mathialagan et al., 2020). The OCTs facilitate the hepatic uptake of cationic drugs (Koepsell, 2021), including the diabetes drug metformin that exhibits higher clearance and decreased systemic exposure during pregnancy in humans (Eyal et al., 2010). In our model, it appeared that PRH regulated OCT1 and OCT3 differently. PRHs increased OCT3 protein concentration across donors at the 10xT3 concentration, but decreased OCT1 expression in a donor-specific manner. These results are consistent with prior reports of modestly suppressed hepatic *Oct1* mRNA and protein levels in pregnant mice, and gestational age-dependent increases in placental human *OCT3* and mouse *Oct3* expression (Lee et al., 2013; Shuster et al., 2013). The PRH triggered increase in OCT3 could be a compensatory mechanism for OCT1 suppression in response to PRHs in SCHH (Vollmar et al., 2017). Because OCT2 mediated renal secretion is a key mediator of metformin clearance, the effect of altered hepatic OCT1 and OCT3 on metformin pharmacokinetics during pregnancy is likely minor. However, reduced function polymorphisms in *OCT1* decrease metformin uptake into hepatocytes and glucose lowering (Shi et al., 2008), and thus the relationship between reduced hepatocyte OCT1 protein concentrations and impaired metformin pharmacodynamics during pregnancy warrants further investigation.

Although the clinical relevance of hepatic P-GP mediated biliary efflux to drug disposition has been questioned (Köck and Brouwer, 2012), multiple P-GP substrates are widely used during pregnancy (Daud et al., 2015). We observed that PRH modestly increased P-GP protein concentration in SCHH at the 10xT3 concentration, and therefore the clinical relevance of this change on biliary efflux *in vivo* may be minimal. In contrast, Han et al. (2020) reported that pregnancy did not alter hepatic protein levels of P-GP in mice (Han et al., 2020). In addition, donor-specific decreases in ENT1, MRP2, and MRP3 protein concentrations were also noteworthy. ENT1 is ubiquitously expressed, plays an active role in the transport of nucleobases, nucleosides, and therapeutic analogues including antiviral and anticancer drugs (Boswell-Casteel and Hays, 2017), and facilitates transfer of the antiviral drug abacavir in placenta (Cerveny et al., 2018). However, pregnancy did not impact abacavir pharmacokinetics (Schalkwijk et al., 2016), and it is not known whether ENT1 mediates abacavir disposition in the liver. MRP2 and MRP3 are biliary and basolateral efflux transport proteins that transport multiple drug substrates into the bile and blood circulation, respectively (Kool et al., 1999; Zhou et al., 2008), including antiviral protease inhibitors (MRP2 substrates) (Andany and Loutfy, 2013) and clopidogrel and acetaminophen conjugates (MRP3 substrates) (Manautou et al., 2005; Ji et al., 2018). Consistent with our results, hepatic Mrp2 and Mrp3 expression was suppressed in pregnant rats (Cao et al., 2001) and pregnant mice (Shuster et al., 2013), respectively. Our results lay the foundation for future studies that investigate the impact of PRHs on the disposition of P-GP, ENT1, MRP2 and MRP3 substrates in SCHH.

Collectively, our results demonstrated that the presence and magnitude of PRH mediated changes in DME and transport protein expression was dependent on protein isoform, PRH concentration, and hepatocyte donor. Although PRHs at T2, T3, T3-90% or 10xT3 significantly impacted one or more proteins in individual donors, a net significant impact across all donors was observed only at the supraphysiological 10xT3 concentration for most proteins. While this is the first study to quantify the impact of PRHs on absolute concentrations of >30 non-CYP DME and transport proteins in multiple female hepatocyte donors, we acknowledge that our study is still limited by the relatively small number (*n* = 5) of donors studied. Differences in basal DME expression and the extent of DME induction across hepatocyte donors are well-documented (Schaefer et al., 2012; MacLean et al., 2017). Inter-donor variations in the basal concentration of DMEs and transport proteins may have influenced presence and magnitude of induction within certain hepatocyte donors and the sensitivity to detect PRH-mediated changes in expression across donors for certain proteins. The inter-donor variability observed in our experimental model could offer potential insight into the high variability of pregnancy-associated changes in clearance that occur *in vivo* with certain drugs (Dallmann et al., 2018a; Mulrenin et al., 2021). In addition, our data with individual hormones suggest that the PRH cocktail effects were primarily driven by P4 and to some extent by E2. However, the PRH cocktail effects were greater in magnitude than observed with the individual PRHs for most proteins, most notably OAT2 and FMO5, suggesting potential additive and antagonistic effects of individual PRHs when administered in combination. In fact, prior studies also pointed out the importance of donor-specific and combinatorial effects of individual PRHs on the expression and function of DMEs in SCHH (Choi et al., 2013; Papageorgiou et al., 2013; Khatri et al., 2021b). Further studies with a larger number of hepatocyte donors are necessary to more precisely quantify additive and antagonistic effects between individual PRHs, and better understand the mechanisms underlying pregnancy associated changes in the hepatic protein concentration and subsequent functional activity of DMEs and transport proteins. In addition, extrahepatic DMEs and transport proteins contribute to total drug disposition; therefore, the presence and extent of PRH-mediated alterations in DME and transport protein expression in other tissues such as intestine and kidney warrants further investigation. Continued improvement in the sensitivity of QTAP methods will allow higher throughput screening in smaller number of hepatocytes, and in other cell types.

## Conclusion

To our knowledge, this study is the first comprehensive evaluation of PRH effects on non-CYP DME and transporter absolute protein concentrations in membrane fractions of SCHH. PRHs impacted the absolute concentration of various non-CYP DMEs and transport proteins in a concentration-, isoform-, and hepatocyte donor-dependent manner. Overall, UGT1A4, CES1, CES2, FMO5, POR, OAT2, OCT3, and P-GP exhibited a significant PRH-evoked net change in expression relative to control across hepatocyte donors, while an additional eight proteins (UGT1A1, UGT2B4, UGT2B10, FMO3, OCT1, MRP2, MRP3, ENT1) exhibited a significant PRH mediated alterations within at least two individual hepatocyte donors. For most proteins, the observed effects were most pronounced and only evident following exposure to the supraphysiologic 10xT3 concentration, and the magnitude of expression changes were 2-fold or less. Therefore, the clinical significance of these effects on drug disposition *in vivo* remain unclear and should be interpreted cautiously. Collectively, these findings provide a foundation for future functional studies focused on PRH-mediated changes in specific hepatic DMEs and transport protein and clinically relevant drug substrates of these pathways prescribed during pregnancy and offer the potential to inform pharmacokinetic models of drug disposition changes in pregnancy.

## Data Availability

The raw data supporting the conclusions of this article will be made available by the authors, without undue reservation.

## Glossary

ABC: ATP-binding cassette
AUC: area under the concentration-time curve
CAR: constitutive androstane receptor
CDCA: Chenodeoxycholic acid (3α,7α-dihydroxy-5β-cholan-24-oic acid)
CES: carboxylesterases
CITCO: 6-(4-Chlorophenyl) Imidazo [2,1-b][1,3] Thiazole-5-Carbaldehyde o-(3,4-dichlorobenzyl) Oxime
CKTL: cocktail
CRT: cortisol
DDF: differential detergent fractionation
DME: drug metabolizing enzyme
DMSO: dimethyl sulfoxide
E1: estrone
E2: estradiol
E3: estriol
EDTA: ethylenediaminetetraacetic acid
ERα: estrogen receptor alpha
FMO: flavin monooxygenases
FXR: farnesoid X receptor
iPRH: individual pregnancy related hormone
LC-MS: liquid chromatography – mass spectrometry
LLOQ: lower limit of quantitation
P4: progesterone
PBPK: physiologically-based pharmacokinetic
PBS: phosphate buffered saline
pGH placental growth hormone: PIPES Piperazine-N,N′-bis(2-ethanesulfonic acid)
PMSF: phenylmethylsulfonyl fluoride
PRH: pregnancy-related hormones
PXR: pregnane X receptor
QTAP: quantitative targeted absolute proteomics
SCHH: sandwich-cultured human hepatocytes
SIL: stable isotope labeled
SLC: solute carrier
T2: trimester 2 average plasma concentration
T3: average trimester 3 average plasma concentration
T3-90%: 90th percentile T3 plasma concentration
10xT3: 10-fold average trimester 3 average plasma concentration
UGT: Uridine 5′-diphospho-glucuronosyltransferase
