# Supplementary material for: Impact of pregnancy related hormones on drug metabolizing enzyme and transport protein concentrations in human hepatocytes
Source: Front Pharmacol. 2022 Sep 21;13:1004010. doi: 10.3389/fphar.2022.1004010 (PMC9532936; doi:10.3389/fphar.2022.1004010)
Supplement: Supplementary file 1 [file DataSheet1.PDF]

Suppl. Table 1. Primary hepatocyte female donor characteristics.

Donors	Age (yr.)	Race	Vendor	Drug history	Cause of Death
**Hu8339**	31	African American	Life Technologies	Marijuana	Asphyxiation
**Hu8373**	26	Caucasian	Life Technologies	Cocaine, cannabis	Asphyxiation
**Hu8375**	19	Caucasian	Life Technologies	Cannabis	Asphyxiation
**YNM**	48	Caucasian	BioIVT	Not reported	Anoxia
**Hu1970**	34	Caucasian	Life Technologies	Not reported	Not reported

Suppl. Table 2. Metabolism of pregnancy related hormones (PRH) in sandwich-cultured human primary hepatocytes (SCHH).

Hormone	Hu8373 ($t_{1/2}$ hr.)	Hu8375 ($t_{1/2}$ hr.)
E2	1.7 ± 0.22	2.1 ± 0.29
E3	1.4 ± 0.05	1.2 ± 0.03
P4	1.3 ± 0.08	1.7 ± 0.12
CRT	>12 hrs.	> 12 hrs.

SCHH medium samples were collected at 0, 8, and 24 hr on experimental day-3 in 2 hepatocyte donors. Media concentration of E2, E3, P4 and CRT in the culture medium was measured in triplicate by ELISA, as described in the methods section. The mean ± SEM elimination half-life ($t_{1/2}$) of each hormone in each donor were calculated (hours). The concentration of E1 and pGH in the medium was not measured due to lack of reliable ELISA kits; thus, the estimated half-life for these PRHs was not calculated.

Suppl. Table 3. Tryptic peptides, their sequences, and SIL peptide MRMs used to report the absolute protein concentration of human liver relevant UGTs and other DMEs in SCHH.

Protein	Peptide sequence	MRM1*	MRM2*
UGT1A1	D$_{70}$GA**F**YTLK$_{77}$	462.75/681.39 (y5)	462.75/524.31 (y4)
UGT1A3	Y$_{164}$LSIPTVFFL**R**$_{174}$	683.39/889.52 (y7)	683.39/1089.63 (y9)
UGT1A4	F$_{74}$FTLTAYAVPWTQ**K**$_{87}$	560.97/667.37 (y5)	840.95/1000.53 (y8)
UGT1A5	Y$_{164}$LSIPAVFFL**R**$_{\mathbf{174}}$	668.39/859.51 (y7)	668.39/1059.62 (y9)
UGT1A6	D$_{44}$IVEVLSD**R**$_{52}$	528.28/599.34 (y5)	528.28/728.38 (y6)
UGT1A7	W$_{98}$TAPL**R**$_{103}$	372.22/466.30 (y4)	372.22/567.35 (y5)
UGT1A8	G$_{52}$HEVVVVMPEVSWQLG**K**$_{68}$	634.67/526.29 (y9)	634.67/720.40 (b7)
UGT1A9	G$_{171}$ILCHYLEEGAQCPAPLSYVP**R**$_{192}$	847.41/1009.56 (y9)	847.41/841.48 (y7)
UGT1A10	Y$_{160}$FSLPSVVFT**R**$_{170}$	663.36/815.47 (y7)	663.36/1015.58 (y9)
UGT2A3	V$_{41}$ILEELIV**R**$_{49}$	547.35/881.53 (y7)	547.35/768.45 (y6)
UGT2B4	F$_{174}$SPGYAIE**K**$_{182}$	510.27/785.42 (y7)	510.27/688.41 (y6)
UGT2B7	A$_{253}$DVWLI**R**$_{259}$	441.76/597.39 (y4)	441.76/696.43 (y5)
UGT2B10	G$_{49}$HEVTVLASSASILFDPNDSSTL**K**$_{72}$	832.77/869.48 (y8)	832.77/1131.54 (y10)
UGT2B15	F$_{175}$SVGYTFE**K**$_{183}$	543.28/752.38 (y6)	543.28/851.44 (y7)
UGT2B17	F$_{175}$SVGYTVE**K**$_{183}$	519.27/803.44 (y7)	519.27/704.37 (y6)
CES1	E$_{394}$LIPEATE**K**$_{402}$	519.29/682.36 (y6)	519.29/341.68 (y6)
CES2	T$_{35}$THTGQVLGSLVHV**K**$_{49}$	396.98/430.78 (y8)	528.97/747.47 (y7)
FMO3	S$_{34}$NDIGGLW**K**$_{42}$	499.26/568.33 (y5)	499.26/796.44 (y7)
FMO5	I$_{293}$ISGLV**K**$_{\mathbf{299}}$	369.25/511.33 (y5)	369.25/624.41 (y6)
POR	G$_{488}$VATNWL**R**$_{495}$	463.76/770.42 (y6)	463.76/598.33 (y4)
γGGT1	L$_{96}$FQPSIQLA**R**$_{105}$	591.85/794.46 (y7)	591.85/389.22 (b3)

**MRMs are roughly in order of highest to lowest intensity.*

Suppl. Table 4. Tryptic peptides, their sequences, and SIL peptide MRMs used to report the absolute protein concentration of human liver relevant transport proteins in SCHH.

Protein / *gene*	Peptide sequence	MRM1*	MRM2*
OATP1A2 / *SLCO1A2*	I$_{591}$YDSTTF**R**$_{598}$	506.75/736.35 (y6)	506.75/621.32 (y5)
OATP1B1 / *SLCO1B1*	N$_{321}$VTGFFQSF**K**$_{330}$	591.81/969.50 (y8)	591.81/868.46 (y7)
OATP1B3 / *SLCO1B3*	I$_{615}$YNSVFFG**R**$_{623}$	556.79/836.43 (y7)	556.79/722.34 (y6)
OATP2B1 / *SLCO2B1*	Y$_{641}$YNNDLL**R**$_{648}$	540.77/754.41 (y6)	540.77/917.47 (y7)
NTCP / *SLC10A1*	G$_{144}$IYDGDL**K**$_{151}$	444.73/718.35 (y6)	444.73/555.29 (y5)
OCT1 / *SLC22A1*	L$_{330}$SPSFADLFR$_{339}$	581.81/481.75(y8)	581.81/865.44(y7)
OCT3 / *SLC22A3*	G$_{522}$IALPETVDDVE**K**$_{534}$	697.37/1039.51 (y9)	697.37/355.23 (b4)
OAT2 / *SLC22A7*	N$_{20}$VALLALP**R**$_{\mathbf{28}}$	488.81/763.51 (y7)	488.81/579.39 (y5)
OAT7 / *SLC22A9*	D$_{313}$TLTLEIL**K**$_{321}$	527.32/724.47 (y6)	527.32/837.55 (y7)
ENT1 / *SLC29A1*	W$_{360}$LPSLVLA**R**$_{368}$	532.83/765.49 (y7)	532.83/383.25 (y7)
ENT2 / *SLC29A2*	S$_{346}$LTSYFLWPDEDS**R**$_{359}$	863.40/728.31 (y6)	863.40/914.39 (y7)
MATE1 / *SLC47A1*	G$_{12}$GPEATLEV**R**$_{\mathbf{21}}$	519.78/462.76 (y8)	519.78/627.37 (y5)
OSTα / *SLC51A*	Y$_{14}$TADLLEVL**K**$_{\mathbf{23}}$	586.84/908.55 (y8)	586.84/837.52 (y7)
OSTβ / *SLC51B*	E$_{73}$TPEVLHLDEA**K**$_{84}$	463.58/579.82 (y10)	463.58/466.77 (y8)
P-GP / *ABCB1*	I$_{368}$IDNKPSIDSYS**K**$_{380}$	496.60/631.32 (y11)	496.60/904.46 (y8)
BSEP / *ABCB11*	S$_{462}$TALQLIQ**R**$_{470}$	520.31/667.41 (y5)	520.31/539.35 (y4)
MRP2 / *ABCC2*	L$_{1377}$TIIPQDPILFSGSL**R**$_{1392}$	890.52/441.31 (b4)	890.52/999.59 (y9)
MRP3 / *ABCC3*	G$_{654}$ALVAVVGPVGCG**K**$_{667}$	646.37/682.35 (y7)	646.37/781.42 (y8
MRP4 / *ABCC4*	E$_{1157}$TIEDLPG**K**$_{1165}$	505.27/666.36 (y6)	505.27/537.31 (y5)
MRP6 / *ABCC6*	T$_{1142}$QAPFVAQNNA**R**$_{1153}$	663.84/301.15 (b3)	663.84/1026.54 (y9)
BCRP / *ABCG2*	S$_{87}$SLLDVLAA**R**$_{96}$	527.81/654.38 (y6)	527.81/767.47 (y7)
Na/K-ATPase / *ATP1A1*	V$_{213}$DNSSLTGESEPQT**R**$_{227}$	543.92/511.29 (y4)	815.38/511.29 (y4)

**MRMs are roughly in order of highest to lowest intensity.*

Suppl. Table 5. Fold-change of 33 DME and transport protein concentrations in sandwich-cultured human hepatocytes (SCHH) exposed to pregnancy related hormone (PRH) cocktails.

**Protein**	**ANOVA P-value***	**T2**		**T3**		**T3-90%**		**10xT3**	
		Mean ±SEM	*P^*	*Mean ±SEM*	*P^*	*Mean ±SEM*	*P^*	*Mean ±SEM*	*P^*
UGT1A1	0.771	1.16 ± 0.10		1.06 ± 0.05		1.14 ± 0.05		1.13 ± 0.17	
UGT1A3	0.865	1.12 ± 0.10		1.01 ± 0.08		1.07 ± 0.09		1.15 ± 0.13	
UGT1A4	<0.001	1.07 ± 0.04	0.277	1.04 ± 0.06	0.517	1.05 ± 0.04	0.414	1.42 ± 0.08	<0.001
UGT1A5	0.198	1.04 ± 0.14		0.91 ± 0.10		1.04 ± 0.17		1.50 ± 0.31	
UGT1A6	0.901	1.12 ± 0.15		1.01 ± 0.10		1.06 ± 0.12		0.97 ± 0.12	
UGT1A9	0.573	1.11 ± 0.09		0.92 ± 0.09		1.04 ± 0.07		1.20 ± 0.18	
UGT2A3	0.448	1.10 ± 0.09		0.92 ± 0.09		0.95 ± 0.06		0.92 ± 0.13	
UGT2B4	0.294	1.12 ± 0.09		0.99 ± 0.03		1.08 ± 0.05		1.26 ± 0.15	
UGT2B7	0.847	1.10 ± 0.07		1.02 ± 0.04		1.05 ± 0.06		1.02 ± 0.11	
UGT2B10	0.242	1.17 ± 0.16		1.11 ± 0.11		1.18 ± 0.08		1.55 ± 0.27	
UGT2B15	0.188	1.24 ± 0.10		1.13 ± 0.04		1.16 ± 0.08		1.01 ± 0.12	
CES1	0.032	1.06 ± 0.09	0.575	1.03 ± 0.05	0.750	0.99 ± 0.04	0.920	1.31 ± 0.11	0.007
CES2	0.017	1.16 ± 0.08	0.277	1.18 ± 0.08	0.215	1.18 ± 0.06	0.219	1.81 ± 0.20	0.001
FMO3	0.104	1.02 ± 0.09		0.94 ± 0.05		0.95 ± 0.09		1.43 ± 0.15	
FMO5	0.002	1.18 ± 0.09	0.074	1.14 ± 0.05	0.128	1.12 ± 0.06	0.200	1.45 ± 0.14	<0.001
POR	0.012	1.11 ± 0.08	0.294	1.07 ± 0.03	0.438	1.16 ± 0.04	0.119	1.52 ± 0.11	0.001
γGGT1	0.442	1.11 ± 0.09		0.99 ± 0.05		1.05 ± 0.08		0.92 ± 0.11	
OAT2	0.001	0.95 ± 0.11	0.627	0.76 ± 0.04	0.063	0.76 ± 0.04	0.060	0.51 ± 0.08	<0.001
OATP1B1	0.273	1.03 ± 0.07		1.01 ± 0.06		1.02 ± 0.06		1.16 ± 0.06	
OATP1B3	0.344	1.06 ± 0.20		0.88 ± 0.10		1.11 ± 0.13		1.26 ± 0.18	
OATP2B1	0.459	1.06 ± 0.21		1.16 ± 0.13		1.07 ± 0.13		0.84 ± 0.22	
OCT1	0.374	1.03 ± 0.08		0.95 ± 0.08		0.94 ± 0.07		0.84 ± 0.13	
OCT3	0.008	1.09 ± 0.15	0.711	1.01 ± 0.09	0.990	1.05 ± 0.15	0.917	1.73 ± 0.24	0.002
NTCP	0.144	1.26 ± 0.10		0.98 ± 0.09		1.07 ± 0.22		0.67 ± 0.17	
ENT1	0.152	0.87 ± 0.14		0.95 ± 0.17		0.84 ± 0.19		0.53 ± 0.10	
OAT7	0.149	1.19 ± 0.07		1.06 ± 0.05		1.15 ± 0.06		1.26 ± 0.11	
OSTα	0.933	1.25 ± 0.31		1.60 ± 0.80		0.93 ± 0.08		1.03 ± 0.21	
MATE1	0.234	0.88 ± 0.16		0.60 ± 0.15		0.90 ± 0.13		0.63 ± 0.22	
MRP3	0.464	1.09 ± 0.20		1.06 ± 0.17		1.03 ± 0.13		0.81 ± 0.13	
BCRP	0.560	1.04 ± 0.20		0.93 ± 0.04		1.07 ± 0.16		1.37 ± 0.33	
BSEP	0.478	0.82 ± 0.08		0.89 ± 0.09		0.95 ± 0.04		0.93 ± 0.12	
MRP2	0.740	1.02 ± 0.13		0.93 ± 0.04		0.94 ± 0.05		0.92 ± 0.15	
P-GP	0.045	1.14 ± 0.13	0.477	1.13 ± 0.06	0.429	1.23 ± 0.11	0.213	1.70 ± 0.28	0.004

The fold change values were calculated relative to the vehicle control group within each hepatocyte donor, and then averaged across the 5 donors.

**The ANOVA p-value for the comparison across the vehicle control and 4 PRH groups is reported. ^For proteins with an ANOVA p < 0.05, the post-hoc Fisher's LSD p-value for the comparison or each PRH group versus vehicle control is reported.*

Supplemental Figure 1.

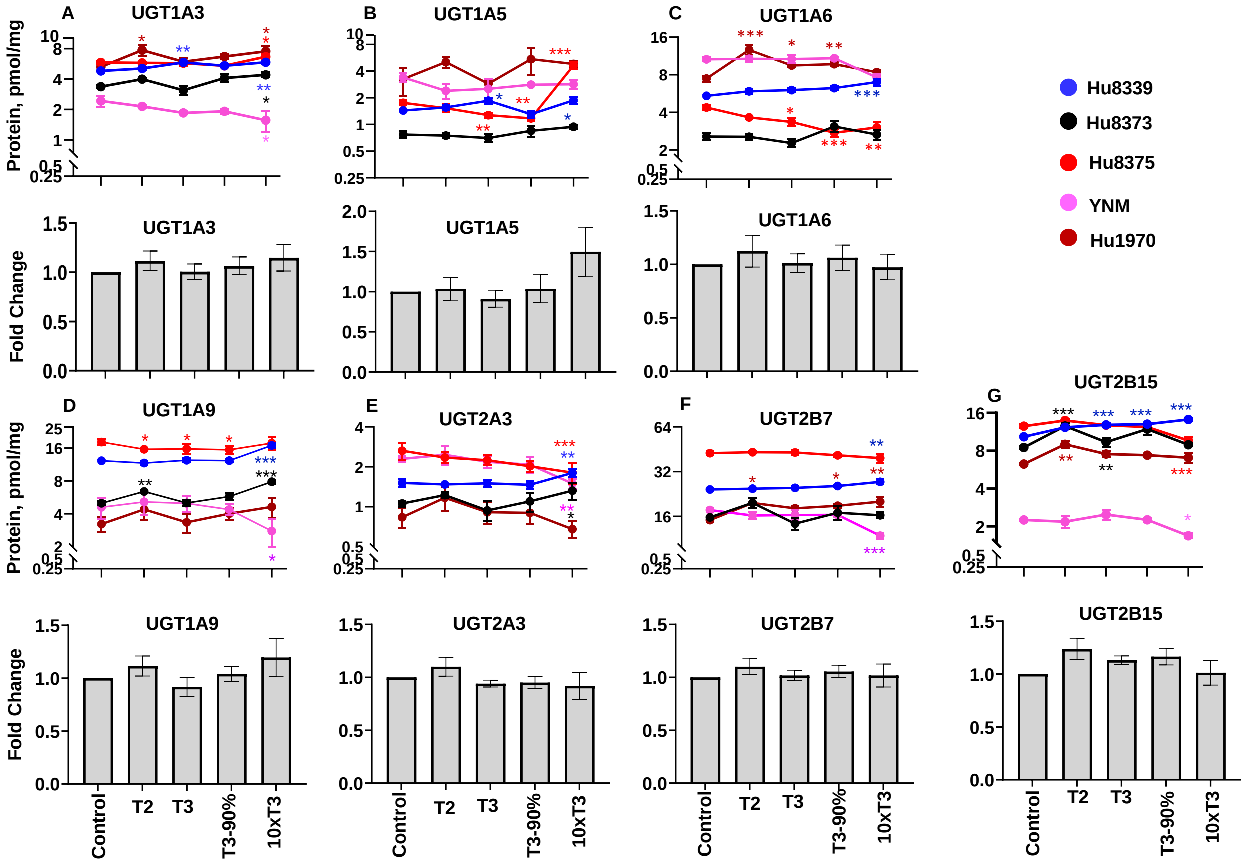

Supplemental Figure 1. *Impact of PRHs on the absolute protein concentration of UGT isoforms in SCHH*. Human primary hepatocytes from 5 qualified donors were exposed to vehicle control or PRH cocktails. The PRH cocktails target average trimester 2 (T2), average trimester 3 (T3), upper range of T3 (T3-90%), and supraphysiological (10xT3) PRH concentrations (Table 1). The line graphs represent mean ± SEM absolute protein concentration of UGT1A3 (**A**), UGT1A5 (**B**), UGT1A6 (**C**), UGT1A9 (**D**), UGT2A3 (**E**), UGT2B7 (**F**), and UGT2B15 (**G**) in SCHH in response to exposure to PRHs within each hepatocyte donor (n=3-4 replicates per group within each donor). The bar graphs below represent the mean ± SEM fold-change for each protein relative to control across all donors (n=5 per group). *p < 0.05, **p < 0.01, ***p < 0.001 versus control.

Supplemental Figure 2.

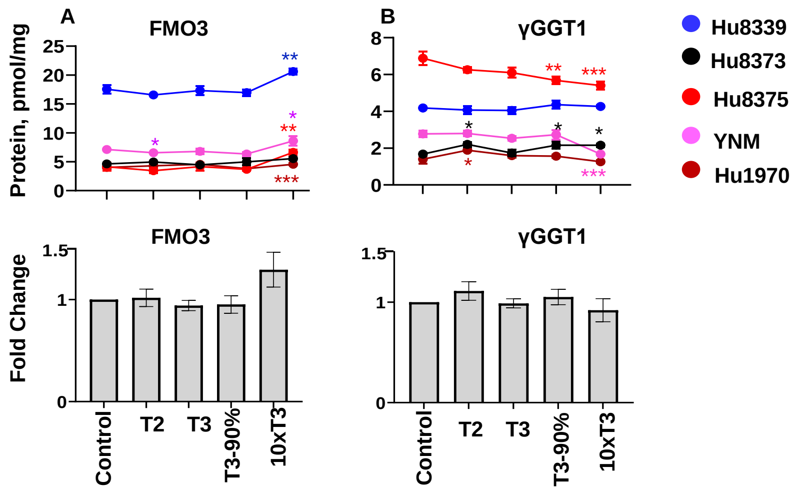

Supplemental Figure 2. *Impact of PRHs on the absolute protein concentration of FMO3 and γGGT1 in SCHH*. Human primary hepatocytes from 5 qualified donors were exposed to vehicle control or PRH cocktails. The PRH cocktails target average trimester 2 (T2), average trimester 3 (T3), upper range of T3 (T3-90%), and supraphysiological (10xT3) PRH concentrations (Table 1). The line graphs represent mean ± SEM absolute protein concentration of FMO3 (**A**) and γGGT1 (**B**) in SCHH in response to exposure to PRHs within each hepatocyte donor (n=3-4 replicates per group within each donor). The bar graphs below represent mean ± SEM fold-change for each protein relative to control across all donors (n=5 per group). *p < 0.05, **p < 0.01, ***p < 0.001 versus control.

Supplemental Figure 3.

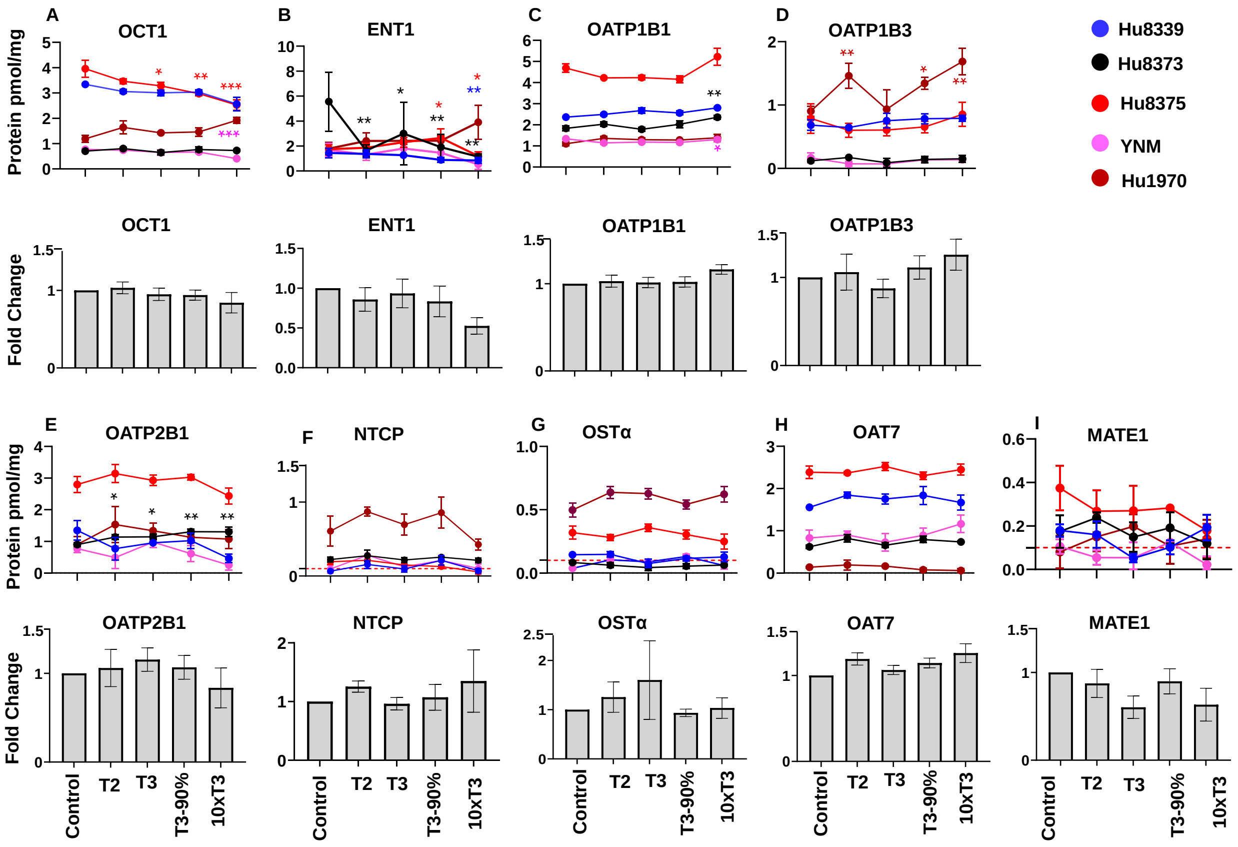

Supplemental Figure 3. *Impact of PRHs on the absolute protein concentration of SLC transport proteins in SCHH*. Human primary hepatocytes from 5 qualified donors were exposed to vehicle control or PRH cocktails. The PRH cocktails target average trimester 2 (T2), average trimester 3 (T3), upper range of T3 (T3-90%), and supraphysiological (10xT3) PRH concentrations (Table 1). The line graphs represent mean ± SEM absolute protein concentration of OCT1 (**A**), ENT1 (**B**), OATP1B1 (**C**), OATP1B3 (**D**), OATP2B1 (**E**), NTCP (**F**), OSTα (**G**), OAT7 (**H**), and MATE (**I**) in SCHH in response to exposure to PRHs within each hepatocyte donor (n=3-4 replicates per group within each donor). The bar graphs below represent mean ± SEM fold-change for each protein relative to control across all donors (n=5 per group). *p < 0.05, **p < 0.01, ***p < 0.001 versus control. p < 0.05, **p < 0.01, ***p < 0.001 versus control.

Supplemental Figure 4.

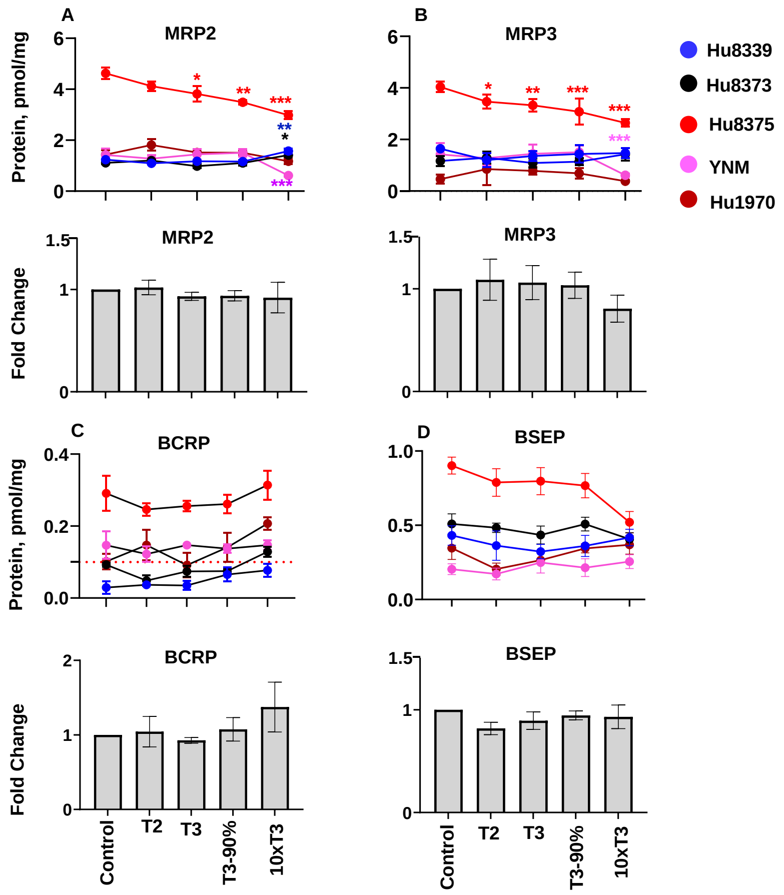

Supplemental Figure 4. *Impacted of PRHs on the absolute protein concentration of ABC transport proteins in SCHH*. Human primary hepatocytes from 5 qualified donors were exposed to vehicle control or PRH cocktails. The PRH cocktails target average trimester 2 (T2), average trimester 3 (T3), upper range of T3 (T3-90%), and supraphysiological (10xT3) PRH concentrations (Table 1). The line graphs represent mean ± SEM absolute protein concentration of MRP2 (**A**), MRP3 (**B**), BCRP (**C**), and BSEP (**D**), in SCHH in response to exposure to PRHs within each hepatocyte donor (n=3-4 replicates per group within each donor). The bar graphs below represent mean ± SEM fold-change for each protein relative to control across all donors (n=5 per group). *p < 0.05, **p < 0.01, ***p < 0.001 versus control. p < 0.05, **p < 0.01, ***p < 0.001 versus control.
